# Association of Medicare Mandatory Bundled Payment System for Hip and Knee Joint Replacement With Racial/Ethnic Difference in Joint Replacement Care

**DOI:** 10.1001/jamanetworkopen.2020.14475

**Published:** 2020-09-22

**Authors:** Hyunjee Kim, Thomas H. A. Meath, Felix W. Tran, Ana R. Quiñones, K. John McConnell, Said A. Ibrahim

**Affiliations:** 1 Center for Health Systems Effectiveness, Oregon Health & Science University, Portland; 2 Department of Family Medicine, Oregon Health & Science University, Portland; 3 Department of Population Health Sciences, Weill Cornell Medicine/New York Presbyterian Health System, New York, New York

## Abstract

**Importance:**

There are marked racial/ethnic differences in hip and knee joint replacement care as well as concerns that value-based payments may exacerbate existing racial/ethnic disparities in care.

**Objective:**

To examine changes in joint replacement care associated with Medicare’s Comprehensive Care for Joint Replacement (CJR) model among White, Black, and Hispanic patients.

**Design, Setting, and Participants:**

Retrospective cohort study of Medicare claims from 2013 through 2017 among White, Black, and Hispanic patients undergoing joint replacement in 67 treatment (selected for CJR participation) and 103 control metropolitan statistical areas.

**Exposures:**

The CJR model holds hospitals accountable for spending and quality of joint replacement care during care episodes (index hospitalization through 90 days after discharge).

**Main Outcomes and Measures:**

The primary outcomes were spending, discharge to institutional postacute care, and readmission during care episodes.

**Results:**

Among 688 346 patients, 442 163 (64.2%) were women, and 87 286 (12.7%) were 85 years or older. Under CJR, spending decreased by $439 for White patients (95% CI, −$718 to −$161; from pre-CJR spending in treatment metropolitan statistical areas of $25 264) but did not change for Black patients and Hispanic patients. Discharges to institutional postacute care decreased for all groups (−2.5 percentage points; 95% CI, −4.7 to −0.4, from pre-CJR risk of 46.2% for White patients; −6.0 percentage points; 95% CI, −9.8 to −2.2, from pre-CJR risk of 59.5% for Black patients; and −4.3 percentage points; 95% CI, −7.6 to −1.0, from pre-CJR risk of 54.3% for Hispanic patients). Readmission risk decreased for Black patients by 3.1 percentage points (95% CI, −5.9 to −0.4, from pre-CJR risk of 21.8%) and did not change for White patients and Hispanic patients. Under CJR, Black-White differences in discharges to institutional postacute care decreased by 3.4 percentage points (95% CI, −6.4 to −0.5, from the pre-CJR Black-White difference of 13.3 percentage points). No evidence was found demonstrating that Black-White differences changed for other outcomes or that Hispanic-White differences changed for any outcomes under CJR.

**Conclusions and Relevance:**

In this cohort study of patients receiving joint replacements, CJR was associated with decreased readmissions for Black patients. Furthermore, Black patients experienced a greater decrease in discharges to institutional postacute care relative to White patients, representing relative improvements despite concerns that value-based payment models may exacerbate existing disparities. Nonetheless, differences between White and Black patients in joint replacement care still persisted even after these changes.

## Key Points

**Question:**

How did joint replacement care change for White, Black, and Hispanic patients under Medicare’s Comprehensive Care for Joint Replacement (CJR) model?

**Findings:**

In this cohort study of 688 346 patients receiving a joint replacement, CJR was associated with a reduction in readmissions for Black patients. Furthermore, whereas Black patients were previously more likely to be discharged to institutional postacute care than White patients, this gap narrowed under CJR.

**Meaning:**

Among patients receiving joint replacements, the reduction in readmissions and decreased discharges to institutional postacute care among Black patients was a positive change under CJR, given concerns that value-based payment models may exacerbate disparities in care for racial/ethnic minorities.

## Teaser

This cohort study analyzes Medicare claims data to assess whether changes in joint replacement care are associated with Medicare’s Comprehensive Care for Joint Replacement (CJR) model among White, Black, and Hispanic patients.

## Introduction

Hip and knee replacements improve function and quality of life for people with severe osteoarthritis, a leading cause of disability among older adults. Hip and knee (“joint” hereafter) replacements are the most common inpatient procedures for Medicare beneficiaries and account for more than 10% of Medicare spending.^1,2^ However, there are marked racial/ethnic differences in surgical outcomes and postsurgical health service use among patients undergoing joint replacement. For example, postsurgery complication is more common for Black patients, contributing to increased readmission and mortality.^3,4,5,6,7,8,9,10,11^ Black patients are more likely to receive institutional postacute care after joint replacement^11,12,13^ and experience less functional improvement in postacute care settings after joint replacement.^14^ Evidence about Hispanic-White differences in joint replacement care is mixed, with some studies reporting similar or lower risk of complications for Hispanic patients than for White patients, while others report higher risk of infections and other complications.^7,15,16,17,18,19^

Medicare value-based payment models, such as bundled payments, aim to reduce spending and improve outcomes for all patients. However, depending on their incentive design, they may have differential effects across racial/ethnic groups. The Comprehensive Care for Joint Replacement (CJR) model, Medicare’s mandatory bundled payment model, was implemented in 67 randomly selected metropolitan statistical areas (MSAs) in April 2016. Under CJR, hospitals are accountable for the spending and quality of care for patients undergoing joint replacement for care episodes that include the index hospitalization through 90 days after hospital discharge. If an episode’s spending exceeds a quality-adjusted spending limit, hospitals are required to repay a portion of the excess spending to Medicare. If the episode spending falls below the CJR limit, hospitals receive a bonus.^2^

However, CJR may differentially affect the care of White, Black, and Hispanic patients receiving joint replacement. In setting CJR spending limits, the Centers for Medicare & Medicaid Services (CMS) does not adjust for patients’ preexisting social or medical complexity, which can affect spending for a joint replacement episode. For example, patients without reliable caregivers or transportation may require institutional postacute care and incur higher costs than patients who go home directly after joint replacement. Socially or medically complex patients are also more likely to experience complications after a surgery, leading to higher readmission rates and higher spending.^18^ Consequently, CJR may incentivize hospitals to avoid admitting potentially more expensive patients, including racial/ethnic minorities, for joint replacements. However, CJR may simultaneously incentivize hospitals to improve care coordination for socially and medically complex patients to decrease spending.^20^ Because Black and Hispanic patients disproportionately constitute socially and medically complex cases, CJR can potentially change care for Black and Hispanic patients more than for White patients and thus decrease racial/ethnic differences in joint replacement care.

Given this potential, we assessed how CJR was associated with changes in joint replacement care among White, Black, and Hispanic patients and with changes in Black-White and Hispanic-White differences in care measures. We hypothesized that CJR was associated with care changes for Black patients and Hispanic patients more than for White patients and therefore reduced Black-White and Hispanic-White differences in joint replacement care. Given the heterogeneity in socioeconomic status within each racial/ethnic group, we also identified patients of low socioeconomic status by their dual Medicaid/Medicare coverage and assessed whether changes in the main analysis were concentrated within low socioeconomic groups of each racial/ethnic group.

## Methods

The institutional review board at Oregon Health & Science University approved this study with a waiver of informed consent because seeking informed consent from all patients included in the study was infeasible and the risk to study participants was minimal. This study followed the Strengthening the Reporting of Observational Studies in Epidemiology (STROBE) reporting guideline.

### Data Sources

We identified joint replacements and subsequent postacute care using the 100% Medicare inpatient, outpatient, skilled nursing facility, home health agency, and carrier claims from years 2013 to 2017. We obtained patient demographic characteristics from the master beneficiary summary files. We used the CMS Provider of Service files and Provider Specific files for hospital and postacute care information (eTable 1 in the Supplement explains variables created from each file).

### Sample Selection

We included inpatient joint replacement surgeries (identified using Medicare Severity Diagnosis Related Group codes 469 and 470) for White, Black, or Hispanic patients in the 67 treatment and 103 control MSAs with admission and discharge dates between January 2013 and June 2015 (pre-CJR) or between April 2016 and September 2017 (post-CJR) (eMethods 1 in the Supplement explains how we selected 67 treatment and 103 control MSAs). We excluded joint replacements occurring from July 2015 to March 2016 because hospitals may have started to change their practice patterns after the CMS announcement of the CJR model in July 2015. We excluded replacements that occurred at hospitals participating in the Bundled Payments for Care Improvement initiative because those hospitals were exempt from CJR. All exclusion criteria are provided in the eFigure in the Supplement.

### Race/Ethnicity

We identified patient race/ethnicity using the Research Triangle Institute race field in the master beneficiary summary files. This field is created by applying an imputation algorithm to the existing race/ethnicity information in the Social Security Administration and Medicare enrollment database. The algorithm uses each beneficiary’s name to identify additional Hispanic or Asian/Pacific Islander beneficiaries. This field is more accurate than race/ethnicity information in the raw enrollment database.^21,22^

### Outcomes

We examined 3 primary outcomes: total episode spending, discharges to institutional postacute care, and relevant readmissions within 90 days of the index hospitalization discharge. We calculated standardized, inflation-adjusted total episode spending as the combined spending of the initial hospitalization and inpatient, outpatient, skilled nursing facility, home health agency, and professional claims occurring during care episodes. We defined discharges to institutional postacute care as discharges in which the patient was sent to a skilled nursing facility, swing bed, inpatient rehabilitation facility, or long-term care hospital. Early evaluations have shown that the use of institutional postacute care decreased under CJR.^23,24,25^ The readmission outcome excludes readmissions for conditions irrelevant to joint replacements. Hospitals participating in CJR were not responsible for spending incurred during these irrelevant readmissions.^2,26^ The list of irrelevant conditions is available on the CMS website.^2^

We also examined secondary outcomes, including discharges to home or home health care, days of institutional postacute care, length of index hospitalization stay, spending incurred at each care setting, postdischarge complication, 90-day emergency department use, and 90-day all-cause mortality.

### Statistical Analysis

We used a difference-in-differences-in-differences framework to assess changes in outcomes associated with CJR for White, Black, and Hispanic patients as well as changes in Black-White and Hispanic-White differences under CJR. All analyses used linear regression models at the episode level. We used linear models for straightforward interpretation of interaction estimates.

The key explanatory variables were (1) the interaction between a treatment MSA measure (ie, whether a joint replacement occurred in treatment MSAs) and a post-CJR measure (ie, whether a joint replacement occurred during the post-CJR period), and (2) 3-way interactions between a treatment MSA measure, a post-CJR measure, and race/ethnicity measures (Black and Hispanic measures with White as the reference group). The first interaction term measured the changes associated with CJR for White patients, while the 3-way interaction terms measured changes in Black-White and Hispanic-White differences under CJR. We calculated the changes under CJR for Black patients and Hispanic patients by summing the coefficients of the first interaction term and those of the 3-way interactions.

Models also included interactions between race/ethnicity and post-CJR measure, interactions between race/ethnicity and treatment MSA measure, race/ethnicity measures, binary measures of each hospital to account for time-invariant hospital characteristics, and binary measures of each year and quarter. Models did not adjust for treatment MSA measure and post-CJR measure separately because they are perfectly collinear with binary measures of hospital and year, respectively. The models are explained in detail in eMethods 2 in the Supplement. Models also adjusted for patient age, sex, and surgery type. We did not adjust for each patient’s Medicaid coverage or baseline health conditions that are potentially highly collinear with race/ethnicity.

As a secondary analysis, we replaced race/ethnicity measures in the main analysis with measures for each race/ethnicity and dual Medicaid/Medicare coverage combination (ie, White non-dual as the reference group, White dual, Black non-dual, Black dual, Hispanic non-dual, and Hispanic dual). We used Medicaid/Medicare coverage to identify patients of low socioeconomic status. Although Black and Hispanic patients disproportionately represent patients of low socioeconomic status, there is significant heterogeneity in socioeconomic status within each racial/ethnic group. This analysis allowed us to assess whether changes in the main analysis were concentrated within low socioeconomic groups within each racial/ethnic group.

We clustered standard errors at the MSA level and applied sampling weights to all analyses to account for oversampling during treatment MSA selection.^27^ We considered *P* values less than .05 significant.

We found no significant differences in pre-CJR trends between treatment and control MSAs for most outcomes across racial/ethnic groups (eTable 2 in the Supplement), supporting the parallel pretrends assumption. Two secondary outcomes (discharge to home and discharge to home health care) for Hispanic patients violated the parallel pretrends assumption, and therefore we did not discuss results for those measures.

We conducted 4 sensitivity analyses. First, we applied propensity score inverse probability weighting to adjust for patient composition differences between treatment and control MSAs (eMethods 3 in the Supplement). Second, we adjusted for baseline health conditions measured by Elixhauser comorbidity index score at admission to assess whether results were explained by health status differences across White patients, Black patients, and Hispanic patients.^28^ Third, we ran models based on the intention-to-treat approach used in prior studies.^23,25^ Finally, we used an instrumental variable approach where we used the initial assignment to CJR as an instrumental variable for the final assignment to CJR (eMethods 4 in the Supplement).^24,25^ All data management was conducted in R, version 3.6.2 (R Core Team), and all statistical analyses were conducted in Stata, version 16 (StataCorp).

## Results

Our sample included 747 098 joint replacement surgeries on 688 346 patients (442 163 [64.2%] were women; 87 286 [12.7%] were 85 years or older). Table 1 displays unadjusted patient and hospital characteristics for joint replacement discharges by patient race/ethnicity during the pre-CJR period. There were 440 555 joint replacements in the pre-CJR period, and 92.9%, 4.3%, and 2.7% of surgeries were for White patients, Black patients, and Hispanic patients, respectively. Black and Hispanic patients were younger than White patients, more likely to have Medicaid coverage, and more likely to be medically complex (defined as a patient in the top decile of the Elixhauser comorbidity index score).

**Table 1.** Unadjusted Patient and Hospital Characteristics for Discharges From Hip or Knee Replacement Surgery, by Patient Race/Ethnicity, Between January 2013 and June 2015

Characteristic	White (weighted N = 416 180)		Black (weighted N = 19 414)		Hispanic (weighted N = 12 426)		Differences by race/ethnicity			
	Weighted No.	Mean (SD), %	Weighted No.	Mean (SD), %	Weighted No.	Mean (SD), %	White vs black, %	*P* value	White vs Hispanic, %	*P* value
**Patient characteristics**										
Age, y										
66-70	115 295	28.2 (0.4)	6774	35.4 (0.5)	3983	32.9 (0.5)	−7.2	<.001	−4.8	<.001
71-75	107 993	26.4 (0.4)	5278	27.6 (0.4)	3370	27.9 (0.4)	−1.2	<.001	−1.5	<.001
76-80	84 895	20.7 (0.4)	3669	19.2 (0.4)	2451	20.3 (0.4)	1.6	<.001	0.5	.31
> 80	101 130	24.7 (0.4)	3430	17.9 (0.4)	2286	18.9 (0.4)	6.8	<.001	5.8	<.001
Female sex	261 703	63.9 (0.5)	13 714	71.6 (0.5)	8025	66.4 (0.5)	−7.7	<.001	−2.4	<.001
Medicaid enrolled	14 862	3.6 (0.2)	3180	16.6 (0.4)	3847	31.8 (0.5)	−13.0	<.001	−28.2	<.001
Medically complex^a^	40 173	9.8 (0.3)	2817	14.7 (0.4)	1437	11.9 (0.3)	−4.9	<.001	−2.1	<.001
Type of joint replacement										
Elective knee	227 427	55.6 (0.5)	11 654	60.9 (0.5)	8346	69.0 (0.5)	−5.3	<.001	−13.5	<.001
Elective hip	119 479	29.2 (0.5)	5032	26.3 (0.4)	2153	17.8 (0.4)	2.9	<.001	11.4	<.001
Hip fracture	62 407	15.2 (0.4)	2465	12.9 (0.3)	1591	13.2 (0.3)	2.4	<.001	2.1	<.001
Major complication or comorbidity	22 542	5.5 (0.2)	1201	6.3 (0.2)	672	5.6 (0.2)	−0.8	<.001	0	.95
**Hospital characteristics**										
Major teaching hospital	87 104	21.3 (0.4)	5813	30.4 (0.5)	2350	19.4 (0.4)	−9.1	<.001	1.8	<.001
Safety-net hospital^b^	10 926	2.7 (0.2)	1784	9.4 (0.3)	1994	16.5 (0.4)	−6.7	<.001	−13.8	<.001
Ownership type										
For profit	70 294	17.2 (0.4)	3135	16.4 (0.4)	2507	20.7 (0.4)	0.8	.003	−3.6	<.001
Not for profit	280 082	68.4 (0.5)	12 715	66.4 (0.5)	7859	65.0 (0.5)	2.0	<.001	3.4	<.001
Public	51 115	12.5 (0.3)	2950	15.4 (0.4)	1551	12.8 (0.3)	−2.9	<.001	−0.3	.32
Other	7823	1.9 (0.1)	352	1.8 (0.1)	174	1.4 (0.1)	0.1	.293	0.5	<.001
Volume of Medicare joint replacements^c^										
Small	15 326	3.7 (0.2)	1553	8.1 (0.3)	1400	11.6 (0.3)	−4.4	<.001	−7.8	<.001
Medium	83 206	20.3 (0.4)	4778	25.0 (0.4)	3372	27.9 (0.4)	−4.6	<.001	−7.6	<.001
High	310 782	75.9 (0.4)	12 820	66.9 (0.5)	7319	60.5 (0.5)	9.0	<.001	15.4	<.001
Mean operating margin^d^	NA	4.5 (14)	NA	2 (17)	NA	1 (19.1)	2.5	<.001	3.5	<.001

Abbreviation: NA, not applicable.

^a^ Medically complex patients are those who were in the top decile of the baseline Elixhauser readmission score.

^b^ Safety-net hospitals were defined as those in the top decile of the Disproportionate Share Hospital index.

^c^ Small-volume, medium-volume, and high-volume hospitals were designated by dividing hospitals into tertiles based on the number of Medicare joint replacements performed annually during the study period.

^d^ Operating margin is defined as the ratio of patient care–related income to patient care–related revenue.

Table 2 and the Figure display changes in primary outcomes associated with CJR for White, Black, and Hispanic patients. Total spending decreased by $439 for White patients (95% CI, −$718 to −$161; from pre-CJR spending in treatment MSAs of $25 264). The spending decrease was not statistically significant for Black patients (−$500; 95% CI, −$1247 to $247) and Hispanic patients (−$419, 95% CI, −$1129 to $291). Discharges to institutional postacute care decreased for all groups, by 2.5 percentage points for White patients (95% CI, −4.7 to −0.4, from the pre-CJR risk of 46.2%), 6.0 percentage points for Black patients (95% CI, −9.8 to −2.2, from the pre-CJR risk of 59.5%), and 4.3 percentage points for Hispanic patients (95% CI, −7.6 to −1.0, from the pre-CJR risk for of 54.3%). Readmission risk decreased by 3.1 percentage points for Black patients (95% CI: −5.9 to −0.4, from the pre-CJR risk of 21.8%), but did not change for White and Hispanic patients.

**Table 2.** Changes in Primary Outcomes Before and After the Comprehensive Care for Joint Replacement Model (CJR) Across White, Black, and Hispanic Patients (N = 747 098)^a^

Outcome	CJR hospitals			Non-CJR hospitals			CJR vs non-CJR hospitals		
	Pre-CJR^b^	Post-CJR^b^	Difference^b^	Pre-CJR^b^	Post-CJR^b^	Difference^b^	Change associated with CJR^c^	95% CI	*P* value
White patients									
Total spending, $	25 264	22 943	−2321	24 613	22 798	−1815	−439	−718 to −161	.002
Discharge to institutional postacute care, %	46.2	33.3	−12.8	45.4	34.9	−10.5	−2.5	−4.7 to −0.4	.02
90-d Readmission, %	16.5	12.1	−4.4	16.0	13.1	−2.9	−1.4	−2.9 to 0.0	.05
Black patients									
Total spending, $	27 600	25 283	−2317	27 096	25 031	−2065	−500	−1247 to 247	.19
Discharge to institutional postacute care, %	59.5	43.9	−15.6	54.3	43.5	−10.7	−6.0	−9.8 to −2.2	.002
90-d Readmission, %	21.8	15.5	−6.3	20.4	16.7	−3.6	−3.1	−5.9 to −0.4	.03
Hispanic patients									
Total spending, $	26 431	24 163	−2268	24 770	23 207	−1563	−419	−1129 to 291	.25
Discharge to institutional postacute care, %	54.3	41.1	−13.2	45.5	36.0	−9.5	−4.3	−7.6 to −1.0	.01
90-d Readmission, %	18.1	14.5	−3.6	17.8	13.9	−3.9	−1.6	−3.7 to 0.6	.15

^a^ All analyses used linear regression models at the episode level and adjusted for the interaction between a treatment metropolitan statistical area (MSA) measure (ie, whether a joint replacement occurred in treatment MSAs) and a post-CJR measure (ie, whether a joint replacement occurred during the post-CJR period), and 3-way interactions between a treatment MSA measure, a post-CJR measure, and race/ethnicity measures (Black and Hispanic measures with White as the reference group). Models also included interactions between race/ethnicity and post-CJR measure, interactions between race/ethnicity and treatment MSA measure, race/ethnicity measures, binary measures of each hospital to account for time-invariant hospital characteristics, and binary measures of each year and quarter. Models also adjusted for patient age, sex, and surgery type.

^b^ Unadjusted values.

^c^ Adjusted values.

**Figure.**
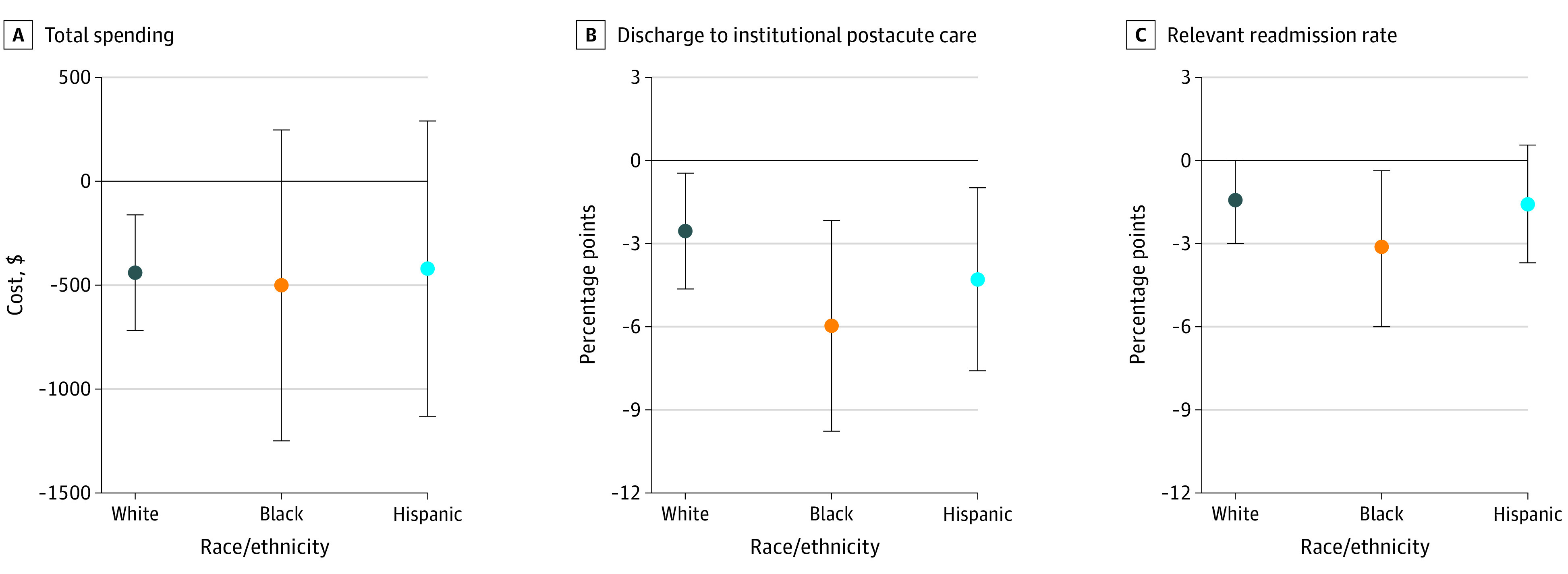
Changes in Primary Outcomes Under the Comprehensive Care for Joint Replacement Model Across White, Black, and Hispanic Patients The bars indicate 95% CIs.

Consistent with decreases in discharges to institutional postacute care, both institutional postacute care spending and the number of days in those institutions decreased in all groups. Despite the reductions in institutional post-acute care, we observed no significant changes in our secondary patient outcomes (emergency department visit, complication, and mortality; eTable 3 in the Supplement).

Table 3 displays changes in black-white and Hispanic-white differences in primary outcomes under CJR (ie, changes in racial/ethnic differences in the treatment MSAs as compared with the corresponding changes in the control MSAs). Black-White differences in discharges to institutional postacute care decreased by 3.4 percentage points (95% CI, −6.4 to −0.5) from the pre-CJR difference of 13.3 percentage points. Similarly, Black-White differences in institutional postacute care spending also decreased by $713 (95% CI, −$1338 to −$87) from the pre-CJR mean of $2381 (eTable 4 in the Supplement). There were no changes in Black-White differences for other outcomes (except a small increase in Black-White differences in discharges to home health) and no changes in Hispanic-White differences for any outcome.

**Table 3.** Changes in Racial/Ethnic Differences for Primary Outcomes Under the Comprehensive Care for Joint Replacement Model (CJR) (N = 747 098)

Outcome	White vs Black				White vs Hispanic			
	Unadjusted difference in treatment MSAs pre-CJR	Adjusted change under CJR^a^	95% CI	*P* value	Unadjusted difference in treatment MSAs pre-CJR	Adjusted change under CJR	95% CI	*P* value
Total spending, $	2336	−61	−704 to 582	.26	1167	20	−637 to 677	.95
Discharge to institutional postacute care, %	13.3	−3.4	−6.4 to −0.5	.02	8.1	−1.7	−4.5 to 1.0	.22
90-d readmission, %	5.3	−1.7	−3.7 to 0.3	.09	1.6	−0.1	−1.9 to 1.6	.87

^a^ “Adjusted change under CJR” measures changes in racial/ethnic differences in the treatment metropolitan statistical areas (MSAs) relative to the corresponding changes in the control MSAs. For example, a −3.4-percentage-point change in adjusted White-Black differences in discharges to institutional postacute care indicates that compared with changes in White-Black differences in the control MSAs, there was 3.5-percentage-point greater reduction in White-Black differences in discharge to institutional postacute care in the treatment MSAs under CJR. All analyses used linear regression models at the episode level and adjusted for the interaction between a treatment MSA measure (ie, whether a joint replacement occurred in treatment MSAs) and a post-CJR measure (ie, whether a joint replacement occurred during the post-CJR period), and 3-way interactions between a treatment MSA measure, a post-CJR measure, and race/ethnicity measures (Black and Hispanic measures with White as the reference group). Models also included interactions between race/ethnicity and post-CJR measure, interactions between race/ethnicity and treatment MSA measure, race/ethnicity measures, binary measures of each hospital to account for time-invariant hospital characteristics, and binary measures of each year and quarter. Models also adjusted for patient age, sex, and surgery type.

Table 4 displays changes in primary outcomes under CJR across racial/ethnic groups combined with dual Medicare/Medicaid coverage. Among Black patients, CJR was associated with decreases in all primary outcomes only for those without Medicaid. Black patients with Medicaid had higher spending, more discharges to institutional postacute care, and more readmissions than Black patients without Medicaid, but we observed no changes among Black patients with Medicaid under CJR.

**Table 4.** Changes in Outcomes Under the Comprehensive Care for Joint Replacement Model (CJR) Across White, Black, and Hispanic Patients by Medicaid Coverage Status (N = 747 098)^a^

Outcome	Unadjusted mean in treatment MSAs pre-CJR	Adjusted change under CJR	95% CI	*P* value	Adjusted difference in changes under CJR	95% CI	*P* value
**White patients**							
Not dual-eligible							
Total spending, $	24 833	−420	−695 to −145	.003	0 [Reference]	NA	NA
Discharge to institutional postacute care, %	44.9	−2.6	−4.8 to −0.4	.02	0 [Reference]	NA	NA
90-d readmission, %	16.1	−1.4	−2.9 to 0.1	.06	0 [Reference]	NA	NA
Dual-eligible							
Total spending, $	35 327	−1040	−2094 to 15	.05	−620	−1662 to 423	.24
Discharge to institutional postacute care, %	76.1	−3.3	−6.1 to −0.5	.02	−0.7	−4.2 to 2.9	.70
90-d readmission, %	24.5	−2.4	−4.5 to −0.2	.03	−0.9	−3.4 to 1.5	.46
**Black patients**							
Not dual-eligible							
Total spending, $	26 200	−812	−1482 to −142	.02	−392	−988 to 203	.20
Discharge to institutional postacute care, %	56.9	−7.0	−11.0 to −3.0	.001	−4.4	−7.5 to −1.3	.005
90-d readmission, %	19.7	−3.0	−5.6 to −0.5	.02	−1.6	−3.6 to 0.3	.10
Dual-eligible							
Total spending, $	33 611	1267	−1016 to 3550	.28	1687	−521 to 3896	.13
Discharge to institutional postacute care, %	70.7	−1.2	−6.4 to 3.9	.64	1.4	−3.4 to 6.1	.57
90-d readmission, %	30.9	−2.9	−9.8 to 3.9	.40	−1.5	−7.7 to 4.7	.63
**Hispanic patients**							
Not dual-eligible							
Total spending, $	24 669	−327	−1067 to 413	.38	93	−569 to 755	.78
Discharge to institutional postacute care, %	47.9	−3.6	−7.4 to 0.3	.07	−1.0	−4.3 to 2.3	.54
90-d readmission, %	16.6	−0.9	−3.6 to 1.9	.53	0.5	−1.8 to 2.9	.66
Dual-eligible							
Total spending, $	29 440	−838	−2054 to 378	.18	−418	−1608 to 712	.50
Discharge to institutional postacute care, %	65.4	−6.6	−11.2 to −1.9	.006	−4.0	−8.5 to 0.6	.09
90-d readmission, %	20.6	−3.5	−6.7 to −0.4	.03	−2.1	−5.2 to 0.9	.18

Abbreviation: NA, not applicable.

^a^ All analyses used linear regression models at the episode level, and adjusted for the interaction between a treatment metropolitan statistical area (MSA) measure (ie, whether a joint replacement occurred in treatment MSAs) and a post-CJR measure (ie, whether a joint replacement occurred during the post-CJR period), and 3-way interactions between a treatment MSA measure, a post-CJR measure, and measures of each race/ethnicity and dual Medicaid/Medicare coverage combination (ie, White non-dual as the reference group, White dual, Black non-dual, Black dual, Hispanic non-dual, and Hispanic dual). Models also included interactions between measures of each race/ethnicity and dual Medicaid/Medicare coverage combination and post-CJR measure, interactions between measures of each race/ethnicity and dual Medicaid/Medicare coverage combination and treatment MSA measure, measures of each race/ethnicity and dual Medicaid/Medicare coverage combination, binary measures of each hospital to account for time-invariant hospital characteristics, and binary measures of each year and quarter. Models also adjusted for patient age, sex, and surgery type.

Models including propensity score weighting (eTable 5 in the Supplement), adjusting for baseline health conditions (eTable 6 in the Supplement), using an intention-to-treat approach (eTable 7 in the Supplement), and using an instrumental variable approach (eTable 8 in the Supplement) demonstrated qualitatively similar results as main results.

## Discussion

Using 100% Medicare claims from 2013 to 2017, we found that discharges to institutional postacute care decreased for White, Black, and Hispanic patients receiving joint replacement under CJR. The decrease in discharges to institutional postacute care was greater for Black patients compared with White patients, thereby decreasing Black-White differences in discharges to institutional postacute care. Despite the reduction in Black patients’ institutional postacute care use, readmissions decreased and other quality measures were unchanged for Black patients. There were no changes in Black-White differences for other outcomes and no changes in Hispanic-White differences for any outcomes under CJR. The decreases in institutional postacute care use and readmissions among Black patients were primarily driven by Black patients without Medicaid coverage.

There is concern that value-based payment models may exacerbate existing disparities.^29,30,31^ If value-based payment models like CJR do not account for patients’ preexisting social or medical complexity, hospitals serving higher proportions of poorer and sicker patients—many of whom are racial/ethnic minority patients—may be more likely to be penalized, thereby increasing racial/ethnic disparities in care. Reinforcing this concern, other studies have found that hospitals serving high proportions of low-income patients under CJR were penalized more than other hospitals.^27,32^ In contrast, our study suggests that despite CJR’s unfavorable design toward hospitals serving high proportions of low-income patients, CJR-participating hospitals may have been able to improve care for Black, but not Hispanic, patients: Black patients’ readmissions decreased despite reductions in their institutional postacute care use.

Compared with White patients, Black patients undergoing joint replacement have had a higher likelihood of institutional postacute care use after surgery.^12^ If higher rates of institutional postacute care use among Black patients reflect more intensive care needs, our findings of decreased discharges to institutional postacute care for Black patients could be concerning. However, previous studies suggest that the use of institutional postacute care has been problematic for Black patients. First, use of institutional postacute care has generally been associated with increased hospital readmissions.^11^ Second, discharges to institutional postacute care, instead of home, are not always shaped by patients’ care needs. In a sample of patients who were potentially dischargeable to home vs an institutional postacute care facility, 38% were discharged to institutional postacute care facilities in the absence of any medical reason.^33,34^ Finally, differences in postacute care expectations (eg, higher likelihood of institutional postacute care use instead of recovering at home) partially shape Black patients’ unwillingness to consider joint replacement surgery.^35,36^

For all of these reasons, our finding of decreased discharges to institutional postacute care for Black patients along with reduced readmissions may be viewed as improvements under CJR. Furthermore, complications, emergency department visits, and mortality did not increase among Black patients under CJR. Nevertheless, readmissions and discharges to institutional postacute care were still more common among Black patients relative to White patients even after these changes under CJR, indicating room for continued improvement. We also need more research to understand whether CJR-participating hospitals reduced use of institutional postacute care for Black patients even when they had medical and social needs for institutional postacute care.

We also examined differences within White, Black, and Hispanic patients according to whether they had Medicaid coverage. We found that changes among Black patients occurred primarily among those without Medicaid, even though Black patients with Medicaid had more discharges to institutional postacute care and readmissions than Black patients without Medicaid. This may indicate challenges in reducing readmissions or discharges to institutional postacute care for Medicaid-enrolled Black patients who may have greater social/medical complexity.

### Limitations

Our study has several limitations. First, the Research Triangle Institute race field captures 77% of patients who self-identified as Hispanic and thus is less reliable than more comprehensive self-reported measures.^37^ However, we used the field because it still outperforms the raw race field in the master beneficiary summary files. Second, CJR-participating hospitals may have avoided admitting potentially more expensive patients (eg, racial/ethnic minorities) for joint replacements. Fewer minority patients may have therefore received joint replacements in treatment MSAs under CJR, making treatment and control MSAs incomparable. To address this limitation, we applied inverse probability of treatment weighting to regression models, and our overall results remained unchanged. However, the inverse probability of treatment weighting only allows us to adjust for patient selection issues driven by factors that can be measured in our data. Future work should examine whether patient selection occurred under CJR. Third, we excluded durable medical equipment and hospice spending in total spending, but they comprised less than 1.0% of total spending and did not change under CJR.^23^ Fourth, functional status and joint pain are meaningful patient-centered outcomes for patients undergoing joint replacement, but we could not measure them using claims data. Finally, our analysis excluded Asian/Pacific Islanders and Native Americans/Alaska Natives, who represented 1% and 0.3% of Medicare joint replacements, respectively.

## Conclusions

We found that discharges to institutional postacute care decreased across White, Black, and Hispanic patients undergoing joint replacement under CJR. We found no evidence to suggest changes in Hispanic-White differences in any outcomes under CJR but did observe a significant decrease in the Black-White difference in discharges to institutional postacute care. The CJR model was associated with a decrease in the readmissions for Black patients; we did not observe changes in other measures of quality of care, despite the substantially reduced use of institutional postacute care. These represent relative improvements, a notable finding given general concerns that value-based payment models may exacerbate disparities in care for racial/ethnic minorities. Nonetheless, racial/ethnic differences in joint replacement care still persist, indicating the need for additional and sustained efforts to create an equitable health care system.

## Notes

Supplement. **eFigure.** Sample selection **eTable 1.** Definition of outcomes and explanatory variables **eTable 2.** Outcomes trends between treatment and control MSAs for white, black, and Hispanic patients during the pre-CJR period **eTable 3.** Changes in secondary outcomes under CJR across White, Black, and Hispanic patients **eTable 4.** Changes in racial/ethnic differences for secondary outcomes under CJR **eTable 5.** Sensitivity analysis – applying propensity score weighting **eTable 6.** Sensitivity analysis – adjusting for baseline health conditions **eTable 7.** Sensitivity analysis – intent-to-treat analysis **eTable 8.** Sensitivity analysis – instrumental variable analysis **eMethods 1.** Study setting **eMethods 2.** Main regression model specification **eMethods 3.** Propensity score calculation and weight application **eMethods 4.** Instrumental variable approach **eReferences.**
