# Supplementary material for: Association of Medicare Mandatory Bundled Payment System for Hip and Knee Joint Replacement With Racial/Ethnic Difference in Joint Replacement Care
Source: JAMA Netw Open. 2020 Sep 22;3(9):e2014475. doi: 10.1001/jamanetworkopen.2020.14475 (PMC7509636; doi:10.1001/jamanetworkopen.2020.14475)
Supplement: Supplement. — eFigure. Sample selection eTable 1. Definition of outcomes and explanatory variables eTable 2. Outcomes trends between treatment and control MSAs for white, black, and Hispanic patients during the pre-CJR period eTable 3. Changes in secondary outcomes under CJR across White, Black, and Hispanic patients eTable 4. Changes in racial/ethnic differences for secondary outcomes under CJR eTable 5. Sensitivity analysis – applying propensity score weighting eTable 6. Sensitivity analysis – adjusting for baseline health conditions eTable 7. Sensitivity analysis – intent-to-treat analysis eTable 8. Sensitivity analysis – instrumental variable analysis eMethods 1. Study setting eMethods 2. Main regression model specification eMethods 3. Propensity score calculation and weight application eMethods 4. Instrumental variable approach eReferences. [file jamanetwopen-e2014475-s001.pdf]

Supplementary Online Content

Kim H, Meath THA, Tran FW, Quiñones AR, McConnell KJ, Ibrahim SA. Association of Medicare mandatory bundled payment system for hip and knee joint replacement with racial/ethnic difference in joint replacement care. *JAMA Netw Open*. 2020;3(9):e2014475. doi:10.1001/jamanetworkopen.2020.14475

eFigure. Sample selection

eTable 1. Definition of outcomes and explanatory variables

eTable 2. Outcomes trends between treatment and control MSAs for white, black, and Hispanic patients during the pre-CJR period

eTable 3. Changes in secondary outcomes under CJR across White, Black, and Hispanic patients

eTable 4. Changes in racial/ethnic differences for secondary outcomes under CJR

eTable 5. Sensitivity analysis – applying propensity score weighting

eTable 6. Sensitivity analysis – adjusting for baseline health conditions

eTable 7. Sensitivity analysis – intent-to-treat analysis

eTable 8. Sensitivity analysis – instrumental variable analysis

eMethods 1. Study setting

eMethods 2. Main regression model specification

eMethods 3. Propensity score calculation and weight application

eMethods 4. Instrumental variable approach

eReferences.

This supplementary material has been provided by the authors to give readers additional information about their work.

© 2020 Kim H et al. *JAMA Network Open.*

eFigure. Sample selection

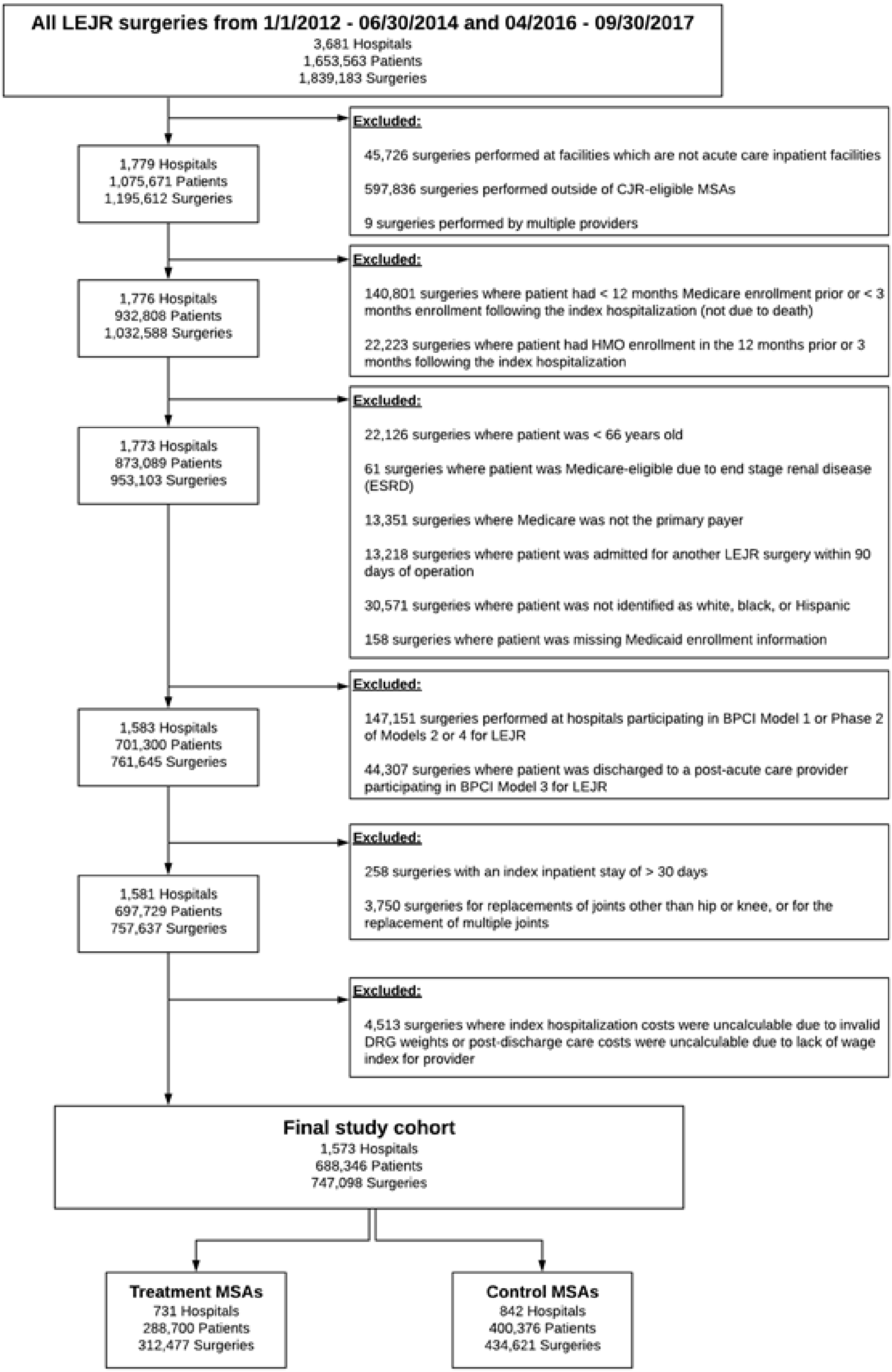

Notes: We identified hospitals participating in BPCI model 1, phase 2 of model 2 or 4 and post-acute care providers participating in BPCI model 3 for joint replacements based on BPCI participant list on the CMS website. We excluded hospitals and post-acute providers from our sample if they ever participated in BPCI models I, phase 2 of model II, model III, and phase 2 of model IV at least once during our study period.

© 2020 Kim H et al. *JAMA Network Open.*

eTable 1. Definition of outcomes and explanatory variables

	Definition	Data source
**Primary outcomes in the main analysis (Table 2-4)**		
Total spending	Standardized, inflation-adjusted Medicare payments that occurred during episode of care (i.e. index LEJR inpatient hospitalization and 90 day post-discharge period) except payments to durable equipment and hospice care	Medicare 100% claims
Discharge to institutional post-acute care	A binary variable that indicates whether a patient was discharged to institutional post-acute settings (inpatient rehabilitation facility, skilled nursing facility, swing bed, and long-term care hospital)	Home Health Agency, Inpatient, and Skilled Nursing Facility Claims
Relevant readmission	A binary variable that indicates whether a patient was readmitted to a hospital within 90 day post-discharge period. We excluded irrelevant readmissions based on CMS definition.	Inpatient Claims and list of MS-DRG and ICD diagnosis codes from CMS[1]
**Secondary outcomes in the main analysis (Table 2-4)**		
**Standardized, inflation-adjusted spending**	We reported all Medicare-allowed payment amounts in 2016 dollars. We also standardized payment amounts by removing Medicare payment variation caused by differences in wage-index, special payments related to medical education, or disproportionate share hospital status.[2]	
Index hospitalization	Medicare payments for index inpatient hospitalization.	Inpatient Claims
Relevant readmission	Medicare payments for relevant readmissions, as defined by CMS, within 90 day post-discharge period.	Inpatient Claims and list of MS-DRG and ICD diagnosis codes from CMS[1]
Institutional post-acute care	Medicare payments for total institutional post-acute care (skilled nursing facility, inpatient rehab, long-term care hospital, and swing bed) within 90 day post-discharge period	Skilled Nursing Facility and Inpatient Claims
Skilled nursing facility	Medicare payments for skilled nursing facility use within 90 day post-discharge period.	Skilled Nursing Facility Claims
Inpatient rehabilitation facility	Medicare payments for inpatient rehabilitation use within 90 day post-discharge period.	Inpatient Claims
Long-term care hospital	Medicare payments for long-term care use within 90 day post-discharge period.	Inpatient Claims
Swing bed	Medicare payments for swing bed care use within 90 day post-discharge period.	Skilled Nursing Facility Claims
Home health agency	Medicare payments for home health care use within 90 day post-discharge period	Home Health Agency Claims
Outpatient facility	Medicare payments for outpatient care use within 90 day post-discharge period.	Outpatient Claims
Professional services	Medicare payments for non-institutional care (e.g. non-institutional physician services) within 90 day post-discharge period.	Medicare Part B Carrier Claims
**Health service use**		
Discharge to home health care	A binary variable that indicates whether a patient was discharged to home health care.	Home Health Agency Claims

© 2020 Kim H et al. *JAMA Network Open.*

Discharge to home	A binary variable that indicates whether a patient was discharged to home without any paid care.	Home Health Agency, Inpatient, and Skilled Nursing Facility Claims
Mean length of stay, Institutional post-acute care facility (days)	Numbers of institutional post-acute care stay days within 90 day post-discharge period.	Skilled Nursing Facility and Inpatient Claims
Mean length of stay, Index hospitalization (days)	Numbers of index hospitalization stay days	Inpatient Claims
**Quality of care**		
Post-discharge complications	A binary variable that indicates whether a patient had any complications within 90 day post-discharge period. We constructed a composite measure of complications that include the occurrence of heart attack, pneumonia, sepsis, surgical site bleeding, pulmonary embolism, mechanical complications, or periprosthetic joint/wound infection following joint replacement. This measure has been modified from NQF #1550 to use our study population as a denominator.	Inpatient and Outpatient Claims and list of MS-DRG and ICD diagnosis codes from NQF[3]
90-day emergency department use	A binary variable that indicates whether a patient visited an emergency department within 90 day post-discharge period.	Inpatient and Outpatient Claims and list of MS-DRG and ICD diagnosis codes from CMS[1]
90-day mortality	A binary variable that indicates whether a patient died during care episode (that includes the index inpatient stay and 90 day post-discharge period).	Inpatient and Outpatient Claims and Master Beneficiary Summary File
**Explanatory variables in the main analysis (Table 2-4)**		
**Key explanatory variables**		
Race/Ethnicity measure	Binary variables that indicates whether a beneficiary was white, black, or Hispanic, with whites as the reference group.	Master Beneficiary Summary File
Treatment MSA measure	A binary variable that indicates whether each hip/knee joint replacement was initiated in one of the MSAs participating in CJR.	Medicare 100% claims and list of hospitals participating in CJR
Post-CJR period measure	A binary variable that indicates whether each hip/knee joint replacement occurred after the implementation of CJR	Inpatient Claims
**Other explanatory variables**		
Surgery-level		
Types of surgery	Binary variables that indicate whether a surgery was hip fracture, elective knee, or elective hip replacement surgery based on CMS definition and ICD-9/-10 codes	Inpatient Claims, CJR Final Rule, and ICD diagnosis codes from CMS[1]
Presence of major complications or comorbidities during hospital stay	A binary variable that indicates whether a patient had any major complications or comorbidities during the hospital stay (MS 469 vs MS 470)	Inpatient Claims
Patient-level		

© 2020 Kim H et al. *JAMA Network Open.*

Age	Binary dummy variables that indicate whether a patient's age was 66-70, 71-75, 76-80, and 81 or above.	Master Beneficiary Summary File
Female	A binary variable that indicates whether a patient was female.	Master Beneficiary Summary File
Binary measure of each hospital	Binary dummy variables that indicate the hospital where hip/knee joint replacements occurred	Inpatient Claims
Binary measure of each year	Binary dummy variables that indicate the year when hip/knee joint replacements occurred	Inpatient Claims
Binary measure of each quarter	Binary dummy variables that indicate the quarter when hip/knee joint replacements occurred	Inpatient Claims
**Hospital-level variables in the descriptive analysis and models with propensity score weighting (Table 1)**		
Major teaching hospital	Binary variable for hospitals with membership in the Council of Teaching Hospitals of the Association of American Medical Colleges.	CMS Provider of Services Files
Safety-net hospital	Binary variable of safety-net hospital status. Safety net hospitals were defined as those in the top decile of disproportionate share hospital index.	CMS Provider of Services Files
Ownership type	Binary variable of hospital ownership type. Non-profit hospitals included those who reported being owned by private non-profits, churches, and other. Public hospitals included those who reported being owned by local, state, or federal government, tribal groups, and hospital districts. This variable was missing for 1% of the hospitals in this study.	CMS Provider of Services Files
Volume of Medicare joint replacements	The number of joint replacement discharges from the hospital during the study period. Joint replacement discharges were identified by applying eligibility criteria from the CJR final rule (CMS, 2015). Low, medium, and high categories were created by breaking volume into tertiles.	Inpatient Claims
Mean operating margin	Continuous measure of operating margin, calculated as the ratio of patient-care-related income to patient-care-related revenue	Healthcare Cost Report Information System

© 2020 Kim H et al. *JAMA Network Open.*

eTable 2. Outcomes trends between treatment and control MSAs for white, black, and Hispanic patients during the pre-CJR period.

	2014			2015		
	**Estimate**	**95% CI**		**Estimate**	**95% CI**	
**White patients**						
Primary outcomes						
Total spending ($)	109	[-150, 368]		52	[-236, 339]	
Discharge to institutional post-acute care (%)	0.2	[-1.2, 1.6]		0.0	[-1.8, 1.9]	
90-day readmission (%)	0.1	[-0.6, 0.8]		0.3	[-0.5, 1.1]	
Secondary outcomes						
Standardized spending ($)						
Index hospitalization	9	[-44, 63]		-15	[-67, 36]	
Readmission	45	[-34, 123]		15	[-85, 114]	
Institutional post-acute care	59	[-140, 258]		162	[-76, 400]	
Long-term care hospital	28	[-3, 59]		33	[-11, 78]	
Inpatient rehabilitation	-17	[-151, 118]		32	[-128, 191]	
Skilled nursing facility	51	[-116, 217]		66	[-113, 244]	
Home health	-15	[-67, 38]		-32	[-111, 46]	
Swing bed	-3	[-47, 41]		31	[-36, 99]	
Outpatient service	-8	[-25, 8]		-5	[-29, 19]	
Physician service	31	[-21, 82]		-7	[-73, 58]	
Health Service Use						
Discharge to home health (%)	-0.1	[-1.9, 1.6]		-0.6	[-3.3, 2.1]	
Discharge to home (%)	-0.1	[-1.5, 1.4]		0.5	[-1.8, 2.9]	
Days in institutional post-acute care	0.2	[-0.2, 0.5]		0.2	[-0.2, 0.6]	
Days in hospital	0.0	[0.0, 0.0]		0.0	[-0.1, 0.0]	
Quality of Care (%)						
Complication	0.0	[-0.3, 0.2]		-0.3	[-0.6, 0.1]	
90-day ED visit	0.2	[-0.4, 0.8]		-0.1	[-0.8, 0.6]	
90-day mortality	0.1	[-0.2, 0.3]		0.0	[-0.3, 0.3]	
**Black patients**						
Primary outcomes						
Total spending ($)	-256	[-1,179, 667]		152	[-889, 1,192]	
Discharge to institutional post-acute care (%)	-2.1	[-5.8, 1.6]		0.2	[-4.4, 4.8]	
90-day readmission (%)	-1.9	[-4.9, 1.1]		-1.9	[-5.1, 1.3]	

© 2020 Kim H et al. *JAMA Network Open.*

Secondary outcomes						
Standardized spending ($)						
Index hospitalization	43	[-97, 183]		49	[-104, 202]	
Readmission	-349	[-791, 93]		-53	[-517, 412]	
Institutional post-acute care	-238	[-1,056, 579]		-362	[-1,298, 573]	
Long-term care hospital	22	[-203, 246]		18	[-199, 235]	
Inpatient rehabilitation	-474	[-1,088, 139]		-625	[-1,296, 46]	
Skilled nursing facility	310	[-262, 881]		438	[-247, 1,123]	
Home health	-26	[-155, 102]		-1	[-184, 182]	
Swing bed	-95	[-316, 126]		-194	[-462, 75]	
Outpatient service	-85	[-171, 2]		-36	[-153,82]	
Physician service	-54	[-186, 77]		-52	[-204, 100]	
Health Service Use						
Discharge to home health (%)	1.2	[-2.2, 4.5]		0.0	[-4.4, 4.3]	
Discharge to home (%)	1.0	[-1.8, 3.7]		-0.2	[-3.7, 3.3]	
Days in institutional post-acute care	0.3	[-1.1, 1.6]		-0.1	[-1.5, 1.3]	
Days in hospital	0.0	[-0.1, 0.1]		0.0	[-0.2, 0.2]	
Quality of Care (%)						
Complication	0.0	[-1.0, 1.0]		0.4	[-1.0, 1.9]	
90-day ED visit	1.6	[-1.4, 4.6]		2.2	[-0.7, 5.1]	
90-day mortality	0.6	[-0.5, 1.6]		0.6	[-0.5, 1.7]	
**Hispanic patients**						
Primary outcomes						
Total spending ($)	206	[-526, 939]		-734	[-1,791, 322]	
Discharge to institutional post-acute care[a] (%)	3.3	[-1.2, 7.8]		-1.7	[-6.5, 3.1]	
90-day readmission (%)	0.2	[-2.6, 3.0]		1.2	[-2.7, 5.1]	
Secondary outcomes						
Standardized spending ($)						
Index hospitalization	-23	[-173, 127]		-115	[-403, 174]	
Readmission	186	[-342, 713]		-117	[-647, 413]	
Institutional post-acute care	290	[-376, 957]		166	[-645, 977]	
Long-term care hospital	183	[-26, 392]		194	[-54, 442]	
Inpatient rehabilitation	143	[-296, 583]		593	[-79, 1,265]	

© 2020 Kim H et al. *JAMA Network Open.*

Skilled nursing facility	-19	[-518, 481]		-677	[-1,382, 28]	
Home health	60	[-60, 179]		57	[-123, 238]	
Swing bed	-17	[-119, 85]		56	[-88, 200]	
Outpatient service	-36	[-137, 65]		-44	[-161, 72]	
Physician service	56	[-115, 228]		105	[-67, 278]	
Health Service Use						
Discharge to home health[a] (%)	0.0	[-4.2, 4.2]		5.7	[0.9, 10.6]	
Discharge to home[a] (%)	-3.3	[-6.1, -0.6]		-4.0	[-8.0, -0.1]	
Days in institutional post-acute care	0.0	[-1.0, 1.0]		-0.8	[-2.2, 0.7]	
Days in hospital	0.1	[-0.1, 0.2]		0.1	[-0.1, 0.3]	
Quality of Care (%)						
Complication	0.2	[-1.1, 1.6]		-0.8	[-2.8, 1.3]	
90-day ED visit	0.5	[-2.3, 3.2]		-2.4	[-5.5, 0.8]	
90-day mortality	-0.2	[-1.6, 1.2]		-1.1	[-2.8, 0.6]	

[a] Outcome violates parallel pre-trend assumption

© 2020 Kim H et al. *JAMA Network Open.*

eTable 3. Changes in secondary outcomes under CJR across White, Black, and Hispanic patients (N=747,098)

	CJR Hospitals				Non-CJR Hospitals				CJR vs. Non-CJR Hospitals		
	(1) Pre-CJR	(2) Post-CJR	(3) Difference		(4) Pre-CJR	(5) Post-CJR	(6) Difference		(7) Change associated with CJR	(8) 95% CI	(9) P-value
**White patients**											
Standardized spending ($)											
Index hospitalization	13,229	12,449	-780		13,132	132,401	-731		-19	[-65, 27]	0.421
Readmission	1382	1291	-92		1,264	1,207	-56		-30	[-89, 29]	0.313
Institutional post-acute care	6149	4255	-1,894		5,941	4,501	-1,439		-450	[-792, -107]	0.010
Long-term care hospital	89	48	-41		110	63	-47		4	[-30, 38]	0.830
Inpatient rehabilitation	1,282	793	-489		1,450	1,040	-410		-82	[-381, 216]	0.587
Skilled nursing facility	4,548	3,239	-1,309		4,093	3,154	-939		-347	[-580, -113]	0.004
Home health	2,023	1,965	-58		1,986	1,951	-35		-3	[-149, 143]	0.969
Swing bed	230	176	-54		288	244	-44		-24	[-70, 22]	0.303
Outpatient service	516	639	123		538	653	116		10	[-15, 34]	0.433
Physician service	3,335	3,185	-151		3,313	3,186	-127		-26	[-80, 28]	0.348
Health Service Use											
Discharge to home health (%)	37.4	42.7	5.3		35.2	38.3	3.2		2.4	[-1.9, 6.6]	0.272
Discharge to home (%)	16.4	23.9	7.5		19.4	26.7	7.3		0.2	[-3.3, 3.7]	0.914
Days in institutional post-acute care	11.8	8.1	-3.7		10.8	8.2	-2.7		-1.0	[-1.5, -0.4]	0.000
Days in hospital	4.3	3.8	-0.6		4.2	3.7	-0.5		0.0	[-0.1, 0.0]	0.356
Quality of Care (%)											
Complication	4.4	3.4	-1.0		3.9	3.0	-0.9		0.1	[-0.2, 0.3]	0.644
90-day ED visit	15.0	18.9	3.9		14.6	18.5	3.9		0.1	[-0.4, 0.6]	0.763
90-day mortality	2.9	2.4	-0.5		2.5	2.1	-0.4		0.0	[-0.1, 0.2]	0.951
**Black patients**											
Standardized spending ($)											
Index hospitalization	13,309	12,563	-746		13,203	12,568	-635		-55	[-160, 50]	0.300
Readmission	1,632	1,784	152		1,681	1,618	-64		156	[-175, 488]	0.354
Institutional post-acute care	8,530	5,938	-2,592		7,952	6,246	-1,706		-1162	[-1,873, -452]	0.001
Long-term care hospital	176	106	-69		289	230	-58		-9	[-154, 137]	0.904

© 2020 Kim H et al. *JAMA Network Open.*

Inpatient rehabilitation	1,987	1,167	-820		1,956	1,568	-389		-481	[-1,117, 154]	0.137
Skilled nursing facility	6,249	4,529	-1,720		5,463	4,128	-1,335		-583	[-1,147, -20]	0.043
Home health	2,273	2,223	-50		2,360	2,248	-112		83	[-148, 314]	0.478
Swing bed	118	136	18		244	319	75		-89	[-352, 175]	0.507
Outpatient service	593	753	160		607	736	128		29	[-34, 93]	0.359
Physician service	3,426	3,296	-131		3,538	3,414	-124		-42	[-159, 76]	0.484
Health Service Use											
Discharge to home health (%)	31.0	40.3	9.3		32.6	36.9	4.3		6.2	[0.8, 11.5]	0.024
Discharge to home (%)	9.5	15.7	6.3		13.1	19.5	6.4		-0.2	[-4.4, 4.1]	0.929
Days in institutional post-acute care	15.9	11.1	-4.8		14.4	10.7	-3.7		-1.6	[-2.8, -0.5]	0.007
Days in hospital	4.7	4.2	-0.5		4.5	3.9	-0.6		0.0	[-0.1, 0.2]	0.495
Quality of Care (%)											
Complication	3.5	3.2	-0.4		3.5	2.9	-0.6		0.0	[-0.8, 0.8]	0.934
90-day ED visit	18.6	23.2	4.6		18.3	23.3	5.0		-0.7	[-2.7, 1.2]	0.465
90-day mortality	2.3	2.1	-0.3		2.4	2.2	-0.2		-0.3	[-0.9, 0.4]	0.451
**Hispanic patients**											
Standardized spending ($)											
Index hospitalization	13,238	12,539	-699		13,153	12,440	-713		64	[-82, 210]	0.388
Readmission	1,449	1,305	-144		1,304	1,311	7		-147	[-491, 197]	0.400
Institutional post-acute care	7,423	5,728	-1,696		6,402	5,069	-1,333		-687	[-1,297, -76]	0.028
Long-term care hospital	187	61	-126		202	154	-49		-63	[-175, 49]	0.267
Inpatient rehabilitation	1,583	1,500	-83		1,863	1,317	-547		-133	[-523, 257]	0.503
Skilled nursing facility	5,580	4,070	-1,510		4,217	3,513	-704		-541	[-1,072, -9]	0.046
Home health	2,286	2,309	22		2,203	2,107	-97		143	[-86, 371]	0.220
Swing bed	74	97	24		119	85	-34		50	[-44, 144]	0.298
Outpatient service	450	621	171		537	668	131		34	[-41, 108]	0.379
Physician service	3,354	3,222	-131		3,237	3,083	-154		-21	[-135, 92]	0.710
Health Service Use											
Discharge to home health[a] (%)	36.2	43.4	7.2		37.5	40.1	2.6		6.0	[1.2, 10.9]	0.015
Discharge to home[a] (%)	9.6	15.6	6.0		17.0	23.9	6.9		-1.7	[-6.3, 2.9]	0.462
Days in institutional post-acute care	14.1	9.8	-4.3		10.9	8.7	-2.2		-1.7	[-2.7, -0.7]	0.001
Days in hospital	4.6	4.0	-0.5		4.2	3.8	-0.5		0.0	[-0.1, 0.1]	0.932

© 2020 Kim H et al. *JAMA Network Open.*

Quality of Care (%)											
Complication	3.8	3.1	-0.6		3.4	2.4	-0.9		0.7	[-0.3, 1.7]	0.165
90-day ED visit	15.8	21.0	5.2		16.6	21.4	4.9		0.1	[-1.6, 1.9]	0.890
90-day mortality	2.3	1.9	-0.4		2.0	1.7	-0.3		0.2	[-0.4, 0.9]	0.468

Notes: [a] Outcome violates parallel pre-trend assumption and should be interpreted with caution. Column (1)-(6) show unadjusted value and Column (7) adjusted value. All analyses used linear regression models at the episode level, and adjusted for the interaction between a treatment MSA measure (i.e., whether a joint replacement occurred in treatment MSAs) and a post-CJR measure (i.e., whether a joint replacement occurred during the post-CJR period), and three-way interactions between a treatment MSA measure, a post-CJR measure, and race/ethnicity measures (black and Hispanic measures with white as the reference group). Models also included interactions between race/ethnicity and post-CJR measure, interactions between race/ethnicity and treatment MSA measure, race/ethnicity measures, binary measures of each hospital to account for time-invariant hospital characteristics, and binary measures of each year and quarter. Models also adjusted for patient age, gender, and surgery type.

© 2020 Kim H et al. *JAMA Network Open.*

eTable 4. Changes in racial/ethnic differences for secondary outcomes under CJR (N=747,098)

	White vs. Black					White vs. Hispanic			
	Unadjusted difference in treatment MSAs pre-CJR	Adjusted change under CJR	95% CI	P-value		Unadjusted difference in treatment MSAs pre-CJR	Adjusted change under CJR	95% CI	P-value
Standardized costs ($)									
Index hospitalization	80	-36	[-135, 62]	0.468		-71	83	[-55, 221]	0.238
Readmission	250	186	[-143, 516]	0.265		-183	-117	[-459, 225]	0.501
Institutional post-acute care	2,381	-713	[-1,338, -87]	0.026		-1,107	-237	[-829, 355]	0.430
Long-term care hospital	86	-13	[-158, 132]	0.864		11	-67	[-179, 46]	0.244
Inpatient rehabilitation	705	-399	[-943, 145]	0.149		-404	-50	[-428, 327]	0.792
Skilled nursing facility	1,701	-236	[-703, 230]	0.318		-669	-194	[-667, 279]	0.420
Home health	250	86	[-64, 236]	0.259		13	145	[-22, 313]	0.088
Swing bed	-112	-65	[-322, 192]	0.621		-44	74	[-31, 179]	0.166
Outpatient service	76	20	[-39, 79]	0.507		-142	24	[-53, 100]	0.539
Physician service	91	-16	[-122, 90]	0.768		-72	5	[-97, 106]	0.930
Health Service Use									
Discharge to home health[a] (%)	-6.5	3.8	[0.1, 7.5]	0.043		5.2	3.7	[0.4, 6.9]	0.027
Discharge to home[a] (%)	-6.9	-0.4	[-3.2, 2.4]	0.786		0.1	-1.9	[-5.3, 1.5]	0.266
Days in institutional post-acute care	4.1	-0.6	[-1.6, 0.3]	0.189		-1.8	-0.7	[-1.6, 0.1]	0.085
Days in hospital	0.3	0.1	[0.0, 0.2]	0.162		-0.1	0.0	[-0.1, 0.1]	0.672
Quality of Care (%)									
Complication	-0.9	-0.1	[-0.9, 0.7]	0.827		0.2	0.6	[-0.3, 1.6]	0.189
90-day ED visit	3.7	-0.8	[-2.8, 1.2]	0.422		-2.8	0.0	[-1.8, 1.8]	0.962
90-day mortality	-0.5	-0.3	[-0.9, 0.4]	0.436		-0.1	0.2	[-0.4, 0.9]	0.476

NOTES: Changes and differences for discharge to home health, discharge to home, complication, 90-day ED visit, and 90-day mortality are listed as percentage-point estimates. [a] Outcome violates parallel pre-trend assumption and should be interpreted with caution

© 2020 Kim H et al. *JAMA Network Open.*

eTable 5. Sensitivity analysis – applying propensity score weighting (N=725,669)

	Treatment vs. Control				Difference in changes associated with CJR (REF: White)		
	Adjusted change associated with CJR	95% CI	P-value		Estimate	95% CI	P-value
**White patients**							
Primary outcomes							
Total spending ($)	-510	[-794, -225]	0.001				
Discharge to institutional post-acute care (%)	-3.1	[-5.4, -0.7]	0.013				
90-day readmission (%)	-1.8	[-3.4, -0.2]	0.029				
Secondary outcomes							
Standardized spending ($)							
Index hospitalization	-27	[-69, 15]	0.202				
Readmission	-41	[-100, 18]	0.171				
Institutional post-acute care	-546	[-902, -189]	0.003				
Long-term care hospital	3	[-29, 35]	0.840				
Inpatient rehabilitation	-147	[-445, 151]	0.332				
Skilled nursing facility	-379	[-624, -135]	0.003				
Home health	-3	[-165, 158]	0.967				
Swing bed	-23	[-71, 26]	0.361				
Outpatient service	3	[-22, 28]	0.802				
Physician service	-39	[-100, 21]	0.202				
Health Service Use							
Discharge to home health (%)	2.8	[-1.8, 7.3]	0.227				
Discharge to home (%)	0.3	[-3.3, 3.8]	0.888				
Days in institutional post-acute care	-1.0	[-1.6, -0.5]	0.001				
Days in hospital	0.0	[-0.1, 0.0]	0.184				
Quality of Care (%)							
Complication	0.1	[-0.2, 0.3]	0.609				
90-day ED visit	-0.1	[-0.6, 0.4]	0.638				
90-day mortality	0.0	[-0.1, 0.2]	0.923				

© 2020 Kim H et al. *JAMA Network Open.*

**Black patients**							
Primary outcomes							
Total spending ($)	-451	[-1,173, 270]	0.219		59	[-572, 689]	0.855
Discharge to institutional post-acute care (%)	-6.7	[-10.6, -2.9]	0.001		-3.7	[-6.5, -0.8]	0.013
90-day readmission (%)	-3.9	[-6.8, -0.9]	0.010		-2.1	[-4.4, 0.2]	0.076
Secondary outcomes							
Standardized spending ($)							
Index hospitalization	-32	[-134, 71]	0.544		-5	[-99, 90]	0.922
Readmission	180	[-142, 501]	0.272		221	[-100, 541]	0.176
Institutional post-acute care	-1,351	[-2,045, -658]	0.000		-806	[-1,419, -193]	0.010
Long-term care hospital	-27	[-178, 124]	0.722		-30	[-178, 11]	0.684
Inpatient rehabilitation	-668	[-1,425, 89]	0.083		-521	[-1,212, 169]	0.138
Skilled nursing facility	-572	[-1,179, 35]	0.065		-192	[-681, 296]	0.438
Home health	69	[-192, 331]	0.602		73	[-81, 226]	0.351
Swing bed	-84	[-320, 152]	0.483		-62	[-292, 168]	0.598
Outpatient service	11	[-57, 79]	0.749		8	[-57, 73]	0.812
Physician service	-23	[-139, 93]	0.693		16	[-89, 122]	0.763
Health Service Use							
Discharge to home health (%)	6.0	[-0.2, 12.2]	0.057		3.2	[-0.8, 7.2]	0.112
Discharge to home (%)	0.7	[-4.2, 5.6]	0.782		0.4	[-2.9, 3.7]	0.795
Days in institutional post-acute care	-1.7	[-2.8, -0.5]	0.006		-0.6	[-1.6, 0.3]	0.187
Days in hospital	0.0	[-0.1, 0.2]	0.493		0.1	[0.0, 0.2]	0.103
Quality of Care (%)							
Complication	-0.2	[-1.0, 0.6]	0.635		-0.3	[-1.1, 0.5]	0.522
90-day ED visit	-0.8	[-2.9, 1.2]	0.428		-0.7	[-2.8, 1.4]	0.499
90-day mortality	-0.5	[-1.2, 0.2]	0.144		-0.5	[-1.2, 0.2]	0.141
**Hispanic patients**							
Primary outcomes							
Total spending ($)	-517	[-1,289, 254]	0.187		-8	[-722, 707]	0.983
Discharge to institutional post-acute care[a] (%)	-4.1	[-7.9, -0.3]	0.033		-1.1	[-4.3, 2.2]	0.529
90-day readmission (%)	-1.4	[-3.6, 0.9]	0.230		0.4	[-1.6, 2.4]	0.679
Secondary outcomes							
Standardized spending ($)							

© 2020 Kim H et al. *JAMA Network Open.*

Index hospitalization	44	[-103, 191]	0.556		71	[-71, 213]	0.325
Readmission	-183	[-489, 123]	0.241		-142	[-447, 164]	0.362
Institutional post-acute care	-680	[-1,405, 46]	0.066		-134	[-857, 589]	0.715
Long-term care hospital	-32	[-151, 87]	0.598		-35	[-149, 79]	0.543
Inpatient rehabilitation	-96	[-610, 419]	0.714		51	[-486, 588]	0.851
Skilled nursing facility	-579	[-1,108, -50]	0.032		-200	[-666, 267]	0.399
Home health	121	[-135, 377]	0.352		125	[-47, 296]	0.153
Swing bed	27	[-67, 121]	0.574		49	[-57, 156]	0.360
Outpatient service	52	[-28, 132]	0.204		49	[-33, 130]	0.240
Physician service	1	[-126, 127]	0.994		40	[-65, 145]	0.454
Health Service Use							
Discharge to home health[a] (%)	5.6	[0.1, 11.1]	0.047		2.8	[-0.8, 6.3]	0.129
Discharge to home[a] (%)	-1.5	[-6.3, 3.4]	0.552		-1.7	[-5.2, 1.8]	0.337
Days in institutional post-acute care	-1.8	[-2.8, -0.7]	0.001		-0.7	[-1.6, 0.1]	0.097
Days in hospital	0.0	[-0.2, 0.1]	0.718		0.0	[-0.1, 0.1]	0.765
Quality of Care (%)							
Complication	0.4	[-0.6, 1.4]	0.385		0.4	[-0.6, 1.4]	0.445
90-day ED visit	0.0	[-2.1, 2.1]	0.988		0.1	[-2.0, 2.3]	0.900
90-day mortality	0.4	[-0.4, 1.1]	0.337		0.4	[-0.4, 1.1]	0.342

Notes: Differences and changes are listed as percentage-point estimates except when specified otherwise.

[a] Outcome violates parallel pre-trend assumption and should be interpreted with caution

© 2020 Kim H et al. *JAMA Network Open.*

eTable 6. Sensitivity analysis – adjusting for baseline health conditions[a] (N=747,098)

	Treatment vs. Control				Difference in changes associated with CJR (REF: White)		
	Adjusted change associated with CJR	95% CI	P-value		Estimate	95% CI	P-value
**White patients**							
Primary outcomes							
Total spending ($)	-469	[-738, -201]	0.001				
Discharge to institutional post-acute care (%)	-2.6	[-4.7, -0.6]	0.013				
90-day readmission (%)	-1.5	[-2.9, -0.1]	0.041				
Secondary outcomes							
Standardized spending ($)							
Index hospitalization	-22	[-68, 25]	0.354				
Readmission	-38	[-95, 20]	0.196				
Institutional post-acute care	-466	[-804, -128]	0.007				
Long-term care hospital	3	[-31, 37]	0.862				
Inpatient rehabilitation	-85	[-384, 213]	0.573				
Skilled nursing facility	-359	[-587, -131]	0.002				
Home health	-3	[-149, 142]	0.963				
Swing bed	-25	[-71, 21]	0.291				
Outpatient service	8	[-16, 32]	0.511				
Physician service	-31	[-83, 22]	0.249				
Health Service Use							
Discharge to home health (%)	2.4	[-1.8, 6.6]	0.257				
Discharge to home (%)	0.2	[-3.3, 3.7]	0.898				
Days in institutional post-acute care	-1.0	[-1.5, -0.5]	0.000				
Days in hospital	0.0	[-0.1, 0.0]	0.299				
Quality of Care (%)							
Complication	0.0	[-0.2, 0.2]	0.764				
90-day ED visit	0.0	[-0.5, 0.5]	0.940				
90-day mortality	0.0	[-0.2, 0.1]	0.894				

© 2020 Kim H et al. *JAMA Network Open.*

**Black patients**							
Primary outcomes							
Total spending ($)	-456	[-1,171, 259]	0.210		14	[-595, 623]	0.964
Discharge to institutional post-acute care (%)	-5.9	[-9.6, -2.2]	0.002		-3.2	[-6.1, -0.4]	0.027
90-day readmission (%)	-3.0	[-5.8, -0.3]	0.030		-1.6	[-3.5, 0.4]	0.120
Secondary outcomes							
Standardized spending ($)							
Index hospitalization	-49	[-153, 55]	0.354		-27	[-125, 70]	0.583
Readmission	171	[-159, 501]	0.308		209	[-119, 536]	0.210
Institutional post-acute care	-1,143	[-1,853, -433]	0.002		-677	[-1,301, -53]	0.034
Long-term care hospital	-7	[-153, 138]	0.921		-10	[-156, 135]	0.888
Inpatient rehabilitation	-478	[-1,117, 160]	0.141		-393	[-939, 153]	0.157
Skilled nursing facility	-569	[-1,112, -16]	0.044		-210	[-667, 248]	0.367
Home health	83	[-149, 314]	0.483		86	[-64, 236]	0.260
Swing bed	-89	[-351, 174]	0.506		-64	[-320, 192]	0.623
Outpatient service	32	[-31, 95]	0.313		24	[-35, 83]	0.420
Physician service	-35	[-148, 79]	0.547		-4	[-107, 99]	0.939
Health Service Use							
Discharge to home health (%)	6.1	[0.8, 11.3]	0.024		3.7	[0.0, 7.3]	0.049
Discharge to home (%)	-0.2	[-4.5, 4.1]	0.929		-0.4	[-3.2, 2.4]	0.767
Days in institutional post-acute care	-1.6	[-2.7, -0.4]	0.006		-0.6	[-1.5, 0.4]	0.231
Days in hospital	0.0	[-0.1, 0.2]	0.437		0.1	[0.0, 0.2]	0.114
Quality of Care (%)							
Complication	0.0	[-0.8, 0.8]	0.990		0.0	[-0.8, 0.7]	0.945
90-day ED visit	-0.6	[-2.5, 1.2]	0.503		-0.7	[-2.6, 1.3]	0.499
90-day mortality	-0.2	[-0.9, 0.4]	0.513		-0.2	[-0.9, 0.4]	0.526
**Hispanic patients**							
Primary outcomes							
Total spending ($)	-435	[-1,140, 269]	0.224		34	[-620, 689]	0.918
Discharge to institutional post-acute care (%)	-4.3	[-7.6, -1.0]	0.011		-1.7	[-4.5, 1.1]	0.241
90-day readmission (%)	-1.6	[-3.8, 0.5]	0.142		-0.1	[-1.9, 1.6]	0.889
Secondary outcomes							
Standardized spending ($)							

© 2020 Kim H et al. *JAMA Network Open.*

Index hospitalization	62	[-82, 206]	0.396		84	[-52, 220]	0.225
Readmission	-152	[-503, 199]	0.393		-114	[-464, 236]	0.520
Institutional post-acute care	-694	[-1,300, -88]	0.025		-228	[-821, 366]	0.450
Long-term care hospital	-63	[-175, 49]	0.265		-66	[-179, 46]	0.247
Inpatient rehabilitation	-134	[-525, 257]	0.500		-49	[-427, 330]	0.801
Skilled nursing facility	-546	[-1,071, -21]	0.042		-187	[-655, 282]	0.432
Home health	142	[-85, 370]	0.219		146	[-21, 313]	0.086
Swing bed	50	[-44, 143]	0.299		74	[-30, 179]	0.163
Outpatient service	33	[-42, 107]	0.391		25	[-52, 101]	0.525
Physician service	-24	[-140, 92]	0.687		7	[-98, 112]	0.897
Health Service Use							
Discharge to home health (%)	6.0	[1.2, 10.8]	0.014		3.6	[0.4, 6.9]	0.029
Discharge to home (%)	-1.7	[-6.3, 2.9]	0.464		-1.9	[-5.3, 1.4]	0.258
Days in institutional post-acute care	-1.7	[-2.7, -0.7]	0.001		-0.7	[-1.6, 0.2]	0.106
Days in hospital	0.0	[-0.1, 0.1]	0.908		0.0	[-0.1, 0.1]	0.659
Quality of Care (%)							
Complication	0.7	[-0.3, 1.6]	0.167		0.6	[-0.3, 1.6]	0.179
90-day ED visit	0.1	[-1.7, 1.9]	0.918		0.1	[-1.8, 1.9]	0.937
90-day mortality	0.2	[-0.4, 0.9]	0.472		0.2	[-0.4, 0.9]	0.453

Notes: Differences and changes are listed as percentage-point estimates except when specified otherwise.

[a] We measured base health conditions by calculating each person's Elixhauser readmission score (using their diagnosis codes during the previous year prior to the index admission) and creating quartiles of the Elixhauser readmission score.

© 2020 Kim H et al. *JAMA Network Open.*

eTable 7. Sensitivity analysis – Intent-to-treat analysis (N=805,546)

	Treatment vs. Control				Difference in changes associated with CJR (REF: White)		
	Adjusted change associated with CJR	95% CI	P-value		Estimate	95% CI	P-value
**White patients**							
Primary outcomes							
Total spending ($)	-357	[-622, -92]	0.008				
Discharge to institutional post-acute care (%)	-2.2	[-4.2, -0.2]	0.032				
90-day readmission (%)	-1.5	[-2.9, -0.2]	0.027				
Secondary outcomes							
Standardized spending ($)							
Index hospitalization	-22	[-55, 10]	0.178				
Readmission	-28	[-83, 28]	0.326				
Institutional post-acute care	-421	[-740, -103]	0.010				
Long-term care hospital	2	[-29,33]	0.885				
Inpatient rehabilitation	-122	[-402, 159]	0.394				
Skilled nursing facility	-291	[-512, -71]	0.010				
Home health	9	[-130, 148]	0.895				
Swing bed	-11	[-59, 38]	0.663				
Outpatient service	10	[-14, 33]	0.420				
Physician service	-25	[-76, 27]	0.351				
Health Service Use							
Discharge to home health (%)	2.5	[-1.5, 6.5]	0.214				
Discharge to home (%)	-0.3	[-3.6, 3.0]	0.848				
Days in institutional post-acute care	-0.8	[-1.4, -0.3]	0.001				
Days in hospital	0.0	[-0.1, 0.0]	0.388				
Quality of Care (%)							
Complication	0.0	[-0.2, 0.2]	0.874				
90-day ED visit	0.1	[-0.4, 0.6]	0.802				
90-day mortality	0.0	[-0.2, 0.1]	0.675				

© 2020 Kim H et al. *JAMA Network Open.*

**Black patients**							
Primary outcomes							
Total spending ($)	-440	[-1,129, 250]	0.210		-82	[-686, 522]	0.789
Discharge to institutional post-acute care (%)	-5.5	[-9.1, -1.9]	0.003		-3.3	[-6.2, -0.5]	0.022
90-day readmission (%)	-2.9	[-5.5, -0.3]	0.030		-1.4	[-3.2, 0.5]	0.160
Secondary outcomes							
Standardized spending ($)							
Index hospitalization	-73	[-126, -20]	0.008		-50	[-102, 1]	0.057
Readmission	146	[-159, 452]	0.346		174	[-130, 477]	0.260
Institutional post-acute care	-1,034	[-1,710, -359]	0.003		-613	[-1,211, -15]	0.045
Long-term care hospital	-8	[-144, 127]	0.902		-11	[-146, 125]	0.875
Inpatient rehabilitation	-438	[-1,026, 149]	0.143		-317	[-821, 188]	0.217
Skilled nursing facility	-504	[-1,032, 25]	0.062		-213	[-651, 226]	0.340
Home health	87	[-135, 310]	0.439		78	[-66, 222]	0.287
Swing bed	-84	[-334, 167]	0.511		-73	[-319, 173]	0.559
Outpatient service	28	[-29, 86]	0.332		19	[-35, 73]	0.494
Physician service	-41	[-155, 72]	0.472		-17	[-117, 83]	0.739
Health Service Use							
Discharge to home health (%)	6.2	[1.2, 11.1]	0.015		3.6	[0.2, 7.1]	0.039
Discharge to home (%)	-0.6	[-4.6, 3.4]	0.754		-0.3	[-3.0, 2.4]	0.817
Days in institutional post-acute care	-1.4	[-2.5, -0.3]	0.011		-0.6	[-1.5, 0.3]	0.215
Days in hospital	0.0	[-0.1, 0.2]	0.416		0.1	[0.0, 0.2]	0.140
Quality of Care (%)							
Complication	-0.1	[-0.9, 0.7]	0.800		-0.1	[-0.9, 0.6]	0.759
90-day ED visit	-0.7	[-2.5, 1.1]	0.429		-0.8	[-2.6, 1.1]	0.401
90-day mortality	-0.2	[-0.9, 0.4]	0.459		-0.2	[-0.9, 0.4]	0.516
**Hispanic patients**							
Primary outcomes							
Total spending ($)	-664	[-1,343, 16]	0.056		-306	[-948, 336]	0.348
Discharge to institutional post-acute care (%)	-4.4	[-7.5, -1.3]	0.006		-2.2	[-4.9, 0.4]	0.100
90-day readmission (%)	-1.8	[-3.8, 0.2]	0.079		-0.3	[-1.9, 1.4]	0.751
Secondary outcomes							
Standardized spending ($)							

© 2020 Kim H et al. *JAMA Network Open.*

Index hospitalization	-6	[-78, 66]	0.871		16	[-55, 88]	0.653
Readmission	-216	[-552, 119]	0.205		-189	[-523, 146]	0.267
Institutional post-acute care	-675	[-1,249, -100]	0.022		-253	[-814, 307]	0.374
Long-term care hospital	12	[-132, 155]	0.873		9	[-137, 155]	0.900
Inpatient rehabilitation	-108	[-504, 288]	0.591		13	[-377, 404]	0.946
Skilled nursing facility	-615	[-1,137, -93]	0.021		-324	[-796, 148]	0.178
Home health	157	[-64, 378]	0.164		147	[-16, 311]	0.076
Swing bed	37	[-50, 123]	0.403		48	[-50, 145]	0.339
Outpatient service	26	[-41, 94]	0.444		17	[-53, 86]	0.634
Physician service	-46	[-152, 60]	0.389		-22	[-117, 73]	0.650
Health Service Use							
Discharge to home health (%)	5.7	[1.1, 10.3]	0.015		3.2	[0.2, 6.2]	0.037
Discharge to home (%)	-1.3	[-5.5, 2.9]	0.543		-1.0	[-4.0, 2.1]	0.538
Days in institutional post-acute care	-1.8	[-2.8, -0.8]	0.000		-1.0	[-1.8, -0.1]	0.022
Days in hospital	-0.1	[-0.2, 0.1]	0.319		0.0	[-0.1, 0.1]	0.497
Quality of Care (%)							
Complication	0.0	[-0.8, 0.7]	0.957		0.0	[-0.8, 0.7]	0.920
90-day ED visit	-0.5	[-2.3, 1.2]	0.539		-0.6	[-2.4, 1.1]	0.495
90-day mortality	-0.1	[-0.7, 0.5]	0.728		-0.1	[-0.7, 0.5]	0.805

Notes: Differences and changes are listed as percentage-point estimates except when specified otherwise. We ran models using 75 treatment and 119 control MSAs, based on the intention-to-treat approach used in prior studies.[4,5]

© 2020 Kim H et al. *JAMA Network Open.*

eTable 8. Sensitivity analysis – Instrumental variable analysis (N=805,546)

	Treatment vs. Control				Difference in changes associated with CJR (REF: White)		
	Adjusted change associated with CJR	95% CI	P-value		Estimate	95% CI	P-value
**White patients**							
Primary outcomes							
Total spending ($)	-406	[-694, 118]	0.006				
Discharge to institutional post-acute care (%)	-2.8	[-5.0, -0.6]	0.014				
90-day readmission (%)	-2.1	[-3.6, -0.5]	0.009				
Secondary outcomes							
Standardized spending ($)							
Index hospitalization	-16	[-61, 29]	0.477				
Readmission	-38	[-98, 22]	0.218				
Institutional post-acute care	-565	[-915, -216]	0.002				
Long-term care hospital	-1	[-33, 30]	0.929				
Inpatient rehabilitation	-215	[-513, 84]	0.157				
Skilled nursing facility	-345	[-586, -103]	0.005				
Home health	25	[-136, 186]	0.761				
Swing bed	-5	[-56, 47]	0.864				
Outpatient service	10	[-15, 35]	0.440				
Physician service	-38	[-95, 20]	0.197				
Health Service Use							
Discharge to home health (%)	3.7	[-0.7, 8.1]	0.099				
Discharge to home (%)	-0.9	[-4.4, 2.7]	0.623				
Days in institutional post-acute care	-1.0	[-1.6, -0.4]	0.001				
Days in hospital	0.0	[-0.1, 0.0]	0.340				
Quality of Care (%)							
Complication	0.0	[-0.2, 0.2]	0.817				
90-day ED visit	0.2	[-0.3, 0.7]	0.524				
90-day mortality	0.0	[-0.2, 0.1]	0.789				

© 2020 Kim H et al. *JAMA Network Open.*

**Black patients**							
Primary outcomes							
Total spending ($)	-316	[-1,060, 427]	0.403		90	[-551, 730]	0.782
Discharge to institutional post-acute care (%)	-6.2	[-10.0, -2.3]	0.002		-3.3	[-6.3, -0.4]	0.029
90-day readmission (%)	-3.4	[-6.3, -0.4]	0.025		-1.3	[-3.4, 0.8]	0.222
Secondary outcomes							
Standardized spending ($)							
Index hospitalization	-28	[-129, 73]	0.586		-12	[-108, 85]	0.810
Readmission	173	[-148, 495]	0.288		211	[-107, 529]	0.192
Institutional post-acute care	-1,130	[-1,862, -398]	0.003		-565	[-1,202, 72]	0.082
Long-term care hospital	-22	[-169, 126]	0.775		-20	[-168, 128]	0.789
Inpatient rehabilitation	-527	[-1,204, 150]	0.126		-313	[-894, 269]	0.290
Skilled nursing facility	-514	[-1,099, 72]	0.085		-169	[-642, 304]	0.482
Home health	114	[-138, 366]	0.374		89	[-64, 242]	0.252
Swing bed	-68	[-306, 171]	0.576		-63	[-299, 173]	0.598
Outpatient service	41	[-20, 103]	0.188		32	[-26, 90]	0.284
Physician service	-36	[-155, 84]	0.559		2	[-104, 108]	0.967
Health Service Use							
Discharge to home health (%)	7.3	[1.7, 12.8]	0.011		3.6	[-0.1, 7.3]	0.059
Discharge to home (%)	-1.1	[-5.5, 3.3]	0.620		-0.2	[-3.1, 2.7]	0.879
Days in institutional post-acute care	-1.5	[-2.7, -0.3]	0.017		-0.5	[-1.4, 0.5]	0.352
Days in hospital	0.1	[-0.1, 0.2]	0.343		0.1	[0.0, 0.2]	0.082
Quality of Care (%)							
Complication	-0.1	[-0.9, 0.7]	0.808		-0.1	[-0.9, 0.6]	0.743
90-day ED visit	-0.6	[-2.6, 1.4]	0.543		-0.8	[-2.8, 1.2]	0.448
90-day mortality	-0.3	[-1.0, 0.4]	0.375		-0.3	[-1.0, 0.4]	0.403
**Hispanic patients**							
Primary outcomes							
Total spending ($)	-566	[-1,355, 222]	0.158		-161	[-896, 575]	0.667
Discharge to institutional post-acute care[a] (%)	-4.8	[-8.2, -1.4]	0.006		-2.0	[-4.8, 0.8]	0.167
90-day readmission (%)	-2.3	[-4.4, -0.1]	0.040		-0.2	[-1.9, 1.5]	0.828
Secondary outcomes							
Standardized spending ($)							

© 2020 Kim H et al. *JAMA Network Open.*

Index hospitalization	89	[-47, 224]	0.199		105	[-24, 234]	0.111
Readmission	-241	[-606, 125]	0.196		-203	[-567, 161]	0.273
Institutional post-acute care	-734	[-1,383, -85]	0.027		-169	[-773, 435]	0.582
Long-term care hospital	20	[-145, 184]	0.811		21	[-144, 187]	0.799
Inpatient rehabilitation	-164	[-617, 290]	0.478		51	[-379, 481]	0.815
Skilled nursing facility	-635	[-1,238, -31]	0.040		-290	[-828, 249]	0.290
Home health	179	[-85, 443]	0.184		154	[-38, 346]	0.116
Swing bed	44	[-51, 139]	0.365		48	[-61, 157]	0.382
Outpatient service	41	[-30, 112]	0.259		31	[-42, 105]	0.403
Physician service	-44	[-158, 71]	0.454		-6	[-104, 93]	0.906
Health Service Use							
Discharge to home health[a] (%)	6.5	[1.3, 11.7]	0.015		2.8	[-0.6, 6.1]	0.104
Discharge to home[a] (%)	-1.7	[-6.1, 2.7]	0.450		-0.8	[-4.1, 2.5]	0.630
Days in institutional post-acute care	-2.0	[-3.0, -0.9]	0.000		-0.9	[-1.8, -0.1]	0.033
Days in hospital	0.0	[-0.2, 0.1]	0.500		0.0	[-0.1, 0.1]	0.802
Quality of Care (%)							
Complication	0.3	[-0.7, 1.4]	0.513		0.3	[-0.7, 1.3]	0.538
90-day ED visit	-0.5	[-2.4, 1.3]	0.571		-0.7	[-2.6, 1.2]	0.462
90-day mortality	0.0	[-0.7, 0.7]	0.982		0.0	[-0.7, 0.7]	0.968

Notes: Differences and changes are listed as percentage-point estimates except when specified otherwise.

© 2020 Kim H et al. *JAMA Network Open.*

eMethods 1. Study Setting

This study included all MSAs that CMS identified as eligible for CJR participation. CMS first excluded 192 of the 388 total MSAs due to high participation rates in the Bundled Payments for Care Improvement (BPCI) Initiative or insufficient volume of joint replacements. The remaining 196 MSAs were then grouped into eight strata based on population size and historical episode spending. CMS randomly selected MSAs for CJR participation within each stratum, oversampling MSAs in strata with higher historical spending. CMS defined the initial sample of 75 treatment (i.e., CJR participation) MSAs and 121 control MSAs in July 2015.[6] However, based on more recent BPCI participation rates during July through September 2015, CMS excluded 8 treatment and 17 control MSAs, leading to 67 treatment MSAs and 104 control MSAs. The final list of treatment and control MSAs is available on the CMS website.[7] We excluded the San Juan MSA from our control group because it was affected by Hurricane Maria in 2017. Our sample contained 67 treatment and 103 control MSAs.

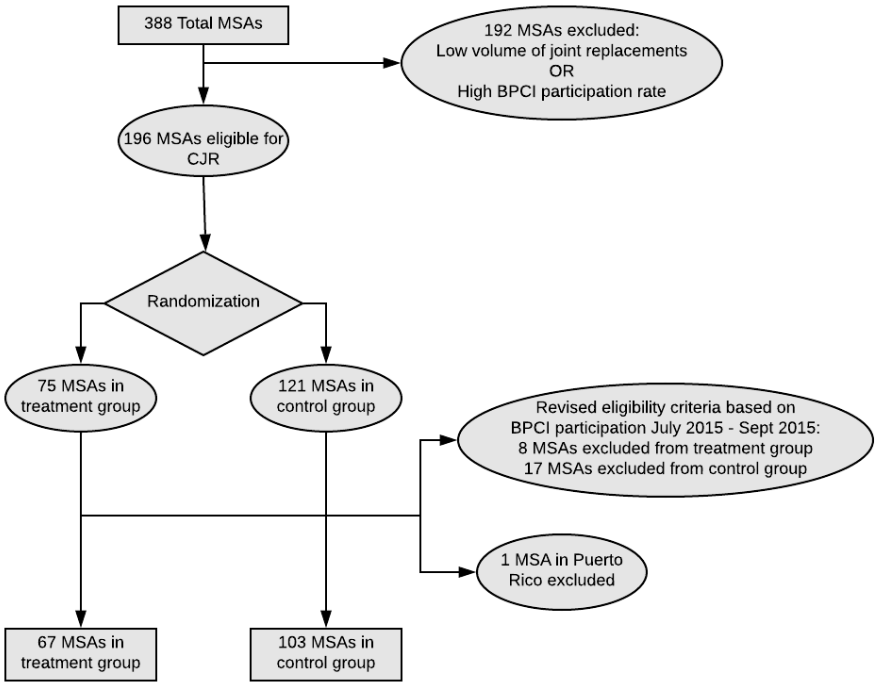

Notes: MSA is metropolitan statistical area, BPCI is Bundled Payment for Care Improvement Initiative, and CJR is Comprehensive Care for Joint Replacement model

© 2020 Kim H et al. *JAMA Network Open.*

eMethods 2. Main regression model specification

The triple-difference approach measures the difference between three estimates derived through the difference-in-differences (DD) approach. In our case, we considered the DD estimate for white, black, and Hispanic patients. The DD estimate for white patients is the average difference (occurring with the CJR implementation) in their outcomes in treatment MSAs subtracted by the average differences in the control MSAs. Likewise, the DD estimates for black and Hispanic patients measure change in outcomes under CJR model among black and Hispanic patients, respectively. The difference between the DD estimates of white, black, and Hispanic patients measures the differential changes across patients with different race/ethnicity under CJR.

More specifically, we estimated the following care-episode-level regression:

$$\begin{aligned} Y_{ihmt} = {} & \beta_0 + \beta_1 Treat_m \times Post_t \times Black_i + \beta_2 Treat_m \times Post_t \times Hispanic_i + \beta_3 Treat_m \times Post_t \\ & + \beta_4 Treat_m \times Black_i + \beta_5 Treat_m \times Hispanic_i + \beta_6 Post_t \times Black_i + \beta_7 Post_t \times Hispanic_i \\ & + \beta_8 Black_i + \beta_9 Hispanic_i + XB + \sum_h \phi_h Hospital_h + \sum_t \gamma_t Year_t + \sum_t \theta_t Quarter_t + \varepsilon_{ihmt} \end{aligned}$$

where Y_{ihmt} is an outcome variable for joint replacement i that occurred in hospital h located in MSA m in year t. $Treat_m$ takes a value of 1 if a joint replacement occurred in the treatment MSAs and 0 otherwise. $Post_t$ takes a value of 1 if a joint replacement occurred after April 2016 and 0 otherwise. $Black_i$ takes a value of 1 for black patients and $Hisapnic_i$ takes a value of 1 for Hispanic patients. X includes patient age groups, gender, and surgery type (elective knee, elective hip, and hip fracture surgery). $Hospital_h$, $Year_t$, and $Quarter_t$ represent binary measures of each hospital, year, and quarter.

β_1 and β_2 measures changes in black-white and Hispanic-white differences under CJR while β_3 measured the changes associated with CJR for white patients.

We obtained changes associated with CJR for black patients by adding β_1 and β_3 (using a post-estimation command that computes linear combinations of coefficient estimates) and changes associated with CJR for Hispanic patients by adding β_2 and β_3. We clustered standard errors on MSAs to account for correlation in error terms within MSAs. Our analysis also included sample weights in regressions to correct for any bias caused by stratified sampling.

© 2020 Kim H et al. *JAMA Network Open.*

eMethods 3. Propensity score calculation and weight application

As a sensitivity analysis, we used propensity score weighting in an attempt to account for differences in hospital and patient composition between treatment and control MSAs. Propensity score weighting, also known as inverse probability of treatment weighting, is a method designed to make observations in the treatment and control groups more comparable by weighing observations based on their probability of being in the treatment group.[8]

Rather than running this method on the entire population, we stratified our study population based on year of joint replacement and race/ethnicity (e.g. white patients with a surgery in 2013, black patients with a surgery in 2017, etc.). We chose to run this process separately for each racial/ethnic group to avoid adjusting out meaningful differences between the racial/ethnic groups. The stratified process instead attempts to make observations in treatment and control MSAs comparable within each racial/ethnic group. We chose to additionally stratify the process by year, as the differences between treatment and control populations may change over time, especially if the CJR program lead to changes in patient composition.

Within each strata, we used patient demographics, hospital characteristics and patient baseline health status to predict whether the surgery occurred within a treatment or control MSA. More specifically, our models contained the following covariates: CMS MSA sampling cluster, hospital volume of joint replacements, hospital bed count, hospital ownership type (public, non-profit, or for-profit), hospital major teaching status, hospital disproportionate patient percentage (DPP), patient gender, patient age, patient Medicaid eligibility, type of joint replacements (elective hip, elective knee, and hip fracture surgery), and Elixhauser mortality and readmission risk scores.

We then used the resulting predictive probability of each surgery occurring in a treatment MSA to calculate Average Treatment Effect (ATE) weights (equation 1).

$$(1) \qquad w_i = \begin{cases} \dfrac{1}{P(Treat_i = 1|X_i)} & where\ Treat = 1 \\ \dfrac{1}{1 - P(Treat_i = 1|X_i)} & where\ Treat = 0 \end{cases}$$

where $P(Treat_i = 1|X_i)$ is the predicted probability that surgery i occurred in a treatment MSA, conditional on covariates X_i. Resulting propensity score weights were multiplied by the CJR sampling weights to create final weights that were applied to all models. We used kernel density plots to confirm that treatment and control groups met the sufficient overlap or common support required for propensity score weighting.

© 2020 Kim H et al. *JAMA Network Open.*

eMethods 4. Instrumental variable approach

As a sensitivity analysis, we used an instrumental variable approach based on previous studies including Finkelstein et al. (2018), Haas et al. (2019), and Barnett et al. (2019).[5,9,10]

Our sample for the instrumental variable analysis included 75 treatment and 121 control MSAs that CMS initially defined for CJR participation in July 2015.[6] We used the initial assignment to CJR (whether an observation was from 75 treatment MSAs versus 121 control MSAs) as an instrumental variable for the final assignment to CJR (whether an observation was from 67 treatment MSAs where CJR was implemented versus other 129 MSAs)

The first stage equation was

$$Treat_m = \alpha_0 + \alpha_1 InitialTreat_m + \sum_k \gamma_k MSA\_Group_m + \epsilon_{ihmt}$$

where $Treat_m$ takes a value of 1 if a joint replacement occurred in the 67 treatment MSAs and 0 otherwise. $InitialTreat_m$ takes a value of 1 if a joint replacement occurred in the 75 treatment MSAs initially defined by CMS and 0 otherwise. MSA_Group_m is eight strata that CMS determined based on population size and historical joint replacement episode spending. CMS randomly selected MSAs for CJR participation within each stratum, oversampling MSAs in strata with higher historical spending.

The second stage equation was

$$\begin{aligned} Y_{ihmt} = {} & \beta_0 + \beta_1 Treat_m \times Post_t \times Black_i + \beta_2 Treat_m \times Post_t \times Hispanic_i + \beta_3 Treat_m \times Post_t \\ & + \beta_4 Treat_m \times Black_i + \beta_5 Treat_m \times Hispanic_i + \beta_6 Post_t \times Black_i + \beta_7 Post_t \times Hispanic_i \\ & + \beta_8 Black_i + \beta_9 Hispanic_i + XB + \sum_h \phi_h Hospital_h + \sum_t \gamma_t Year_t + \sum_t \theta_t Quarter_t + \varepsilon_{ihmt} \end{aligned}$$

© 2020 Kim H et al. *JAMA Network Open.*

eReferences

1. Center for Medicare and Medicaid Services. Comprehensive Care for Joint Replacement (CJR) Model. (2015).
2. Kim, H. *et al.* Association of the Mandatory Medicare Bundled Payment With Joint Replacement Outcomes in Hospitals With Disadvantaged Patients. *JAMA Netw. Open* **2**, e1914696–e1914696 (2019).
3. Center for Medicare and Medicaid Services. Bundled Payments for Care Improvement (BPCI) Initiative: General Information. https://innovation.cms.gov/innovation-models/bundled-payments (2020).
4. Barnett, M. L. *et al.* Two-Year Evaluation of Mandatory Bundled Payments for Joint Replacement. *N. Engl. J. Med.* **0**, null (2019).
5. Haas, D. A., Zhang, X., Kaplan, R. S. & Song, Z. Evaluation of Economic and Clinical Outcomes Under Centers for Medicare & Medicaid Services Mandatory Bundled Payments for Joint Replacements. *JAMA Intern. Med.* **179**, 924–931 (2019).
6. Centers for Medicare and Medicaid Services. *Federal Register: Medicare Program; Comprehensive Care for Joint Replacement Payment Model for Acute Care Hospitals Furnishing Lower Extremity Joint Replacement Services; Final Rule*. https://www.gpo.gov/fdsys/pkg/FR-2015-11-24/pdf/2015-29438.pdf (2015).
7. Centers for Medicare and Medicaid Services. Comprehensive Care for Joint Replacement Model. https://innovation.cms.gov/initiatives/cjr (2019).
8. Stuart, E. A. *et al.* Using propensity scores in difference-in-differences models to estimate the effects of a policy change. *Health Serv. Outcomes Res. Methodol.* **14**, 166–182 (2014).
9. Finkelstein, A., Ji, Y., Mahoney, N. & Skinner, J. Mandatory Medicare Bundled Payment Program for Lower Extremity Joint Replacement and Discharge to Institutional Postacute Care: Interim Analysis of the First Year of a 5-Year Randomized Trial. *JAMA* **320**, 892–900 (2018).
10. Barnett, M. L. *et al.* Two-Year Evaluation of Mandatory Bundled Payments for Joint Replacement. *N. Engl. J. Med.* **380**, 252–262 (2019).

© 2020 Kim H et al. *JAMA Network Open.*
